# The Role of Killer Ig-like Receptors in Diseases from A to Z

**DOI:** 10.3390/ijms26073242

**Published:** 2025-03-31

**Authors:** Luisa Agnello, Anna Masucci, Martina Tamburello, Roberta Vassallo, Davide Massa, Rosaria Vincenza Giglio, Mauro Midiri, Caterina Maria Gambino, Marcello Ciaccio

**Affiliations:** 1 Institute of Clinical Biochemistry, Clinical Molecular Medicine, and Clinical Laboratory Medicine, Department of Biomedicine, Neurosciences and Advanced Diagnostics, University of Palermo, 90133 Palermo, Italy; luisa.agnello@unipa.it (L.A.); anna.masucci@unipa.it (A.M.); martina.tamburello@unipa.it (M.T.); roberta.vassallo03@unipa.it (R.V.); davide.massa@unipa.it (D.M.); rosariavincenza.giglio@unipa.it (R.V.G.); caterinamaria.gambino@unipa.it (C.M.G.); 2 Department of Laboratory Medicine, University Hospital “P. Giaccone”, 90127 Palermo, Italy; 3 Institute of Legal Medicine, Department of Health Promotion, Mother and Child Care, Internal Medicine and Medical Specialties, University of Palermo, 90133 Palermo, Italy; mauro.midiri@unipa.it

**Keywords:** KIR, autoimmune diseases, polymorphisms, genetic

## Abstract

Killer Ig-like Receptors (KIRs) regulate immune responses, maintaining the balance between activation and inhibition of the immune system. KIRs are expressed on natural killer cells and some CD8 T cells and interact with HLA class I molecules, influencing various physiological and pathological processes. KIRs’ polymorphism creates a variability in immune responses among individuals. KIRs are involved in autoimmune disorders, cancer, infections, neurological diseases, and other diseases. Specific combinations of KIRs and HLA are linked to several diseases’ susceptibility, progression, and outcomes. In particular, the balance between inhibitory and activating KIRs can determine how the immune system responds to pathogens and tumors. An imbalance can lead to an excessive response, contributing to autoimmune diseases, or an inadequate response, allowing immune evasion by pathogens or cancer cells. The increasing number of studies on KIRs highlights their essential role as diagnostic and prognostic biomarkers and potential therapeutic targets. This review provides a comprehensive overview of the role of KIRs in all clinical conditions and diseases, listed alphabetically, where they are analyzed.

## 1. Introduction

Killer cell immunoglobulin-like receptors (KIRs) are a family of transmembrane glycoproteins expressed on the surface of natural killer (NK) cells and a subset of CD8 T lymphocytes. They play a crucial role in regulating immune responses by interacting with specific human leukocyte antigen (HLA) class I molecules.

The polygenic nature of the KIR locus, combined with the diversity of their HLA ligands, contributes to the complexity of their function because KIR genes encode receptors that can either inhibit or activate their target cells [1].

Combinations of KIR alleles and HLA ligands are associated with several diseases, such as autoimmune disorders, infectious diseases, cancer, and transplantation.

**Figure 1:**
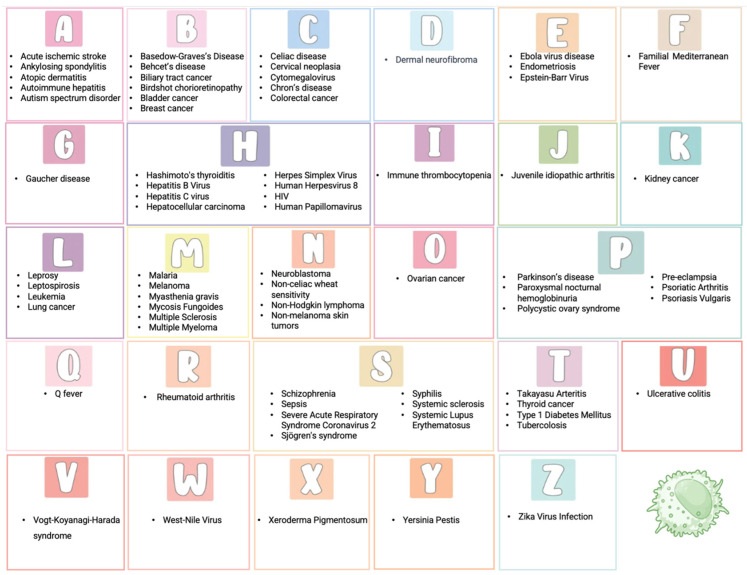
Role of KIR genes in diseases: an alphabetical overview.

This review aims to provide a comprehensive overview of the involvement of KIRs in diseases (Figure 1). We will examine current research and clinical findings to explore how KIRs impact disease susceptibility, progression, and therapeutic responses. Understanding these mechanisms may offer novel insights into potential diagnostic markers and therapeutic targets, ultimately contributing to improved disease management and treatment strategies.

## 2. Natural Killer Cells

**Figure 2:**
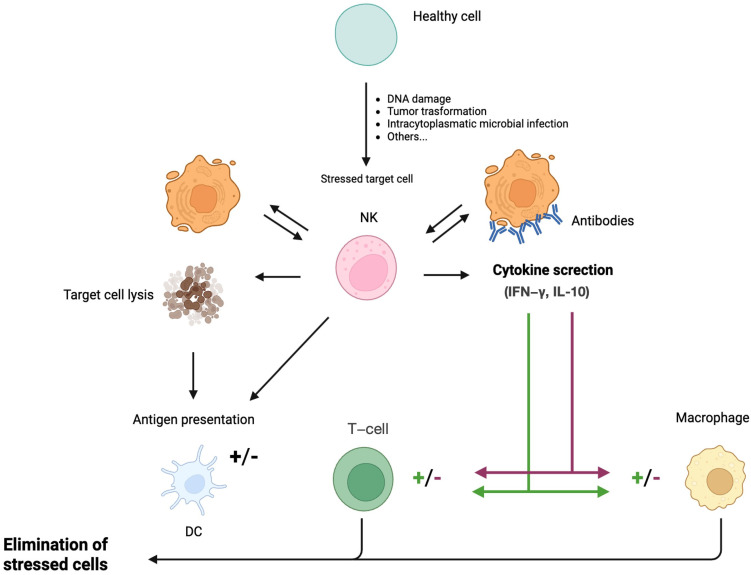
Biological functions of NK cells. NK cells recognize stressed cells (transformed or virus-infected cells) in the presence or absence of antibodies. Recognition ultimately leads to the elimination of the stressed cell and the production of cytokines by NK cells. NK cells can interact with DCs by killing immature DCs and promoting DC maturation through the release of IFN-γ and TNF-α, resulting in enhanced antigen presentation to T cells. Furthermore, NK cells modulate macrophage and T-cell responses by releasing IFN-γ and TNF-α, either potentiating or attenuating the respective immune reactions. NK: Natural killer cell; DC: Dendritic cell; IFN-γ: Interferon-γ; IL-10: Interleukin-10.

NK cells are effector lymphocytes, accounting for approximately 10% of lymphocytes in human peripheral blood [2]. They belong to the innate immune system but can also participate in the acquired immune response through cytotoxic activity and cytokine production [3] (Figure 2).

A balance of intracellular signals from activating and inhibitory receptors regulates NK cells’ functions, which recognize specific ligands on target cells. Activating receptors recognize ligands that are expressed de novo on stressed cells or are overexpressed in tumor or virus-infected cells [4], inducing apoptosis of the target cell through secretion of perforin and granzymes or engagement of receptor-mediated apoptosis by expressing tumor necrosis factor-related apoptosis-inducing ligand, as well as Fas ligand [5]. Moreover, activated NK cells can secrete cytokines, like interferon-γ (IFN-γ), tumor necrosis factor-α (TNF-α), and granulocyte-macrophage colony-stimulating factors, which activate both innate and adaptive immune cells.

Human NK cells express two major activating receptor classes: the natural cytotoxicity receptor and Natural-killer group 2, member D (NKG2D).

Inhibitory receptors include members of KIRs (expressed only in humans) and the CD94-NKG2A heterodimer (expressed in both mice and humans). They are necessary to prevent NK-mediated autoreactivity by providing “off” signals without impairing the capacity of NK cells to respond to other stimuli [6].

**Figure 3:**
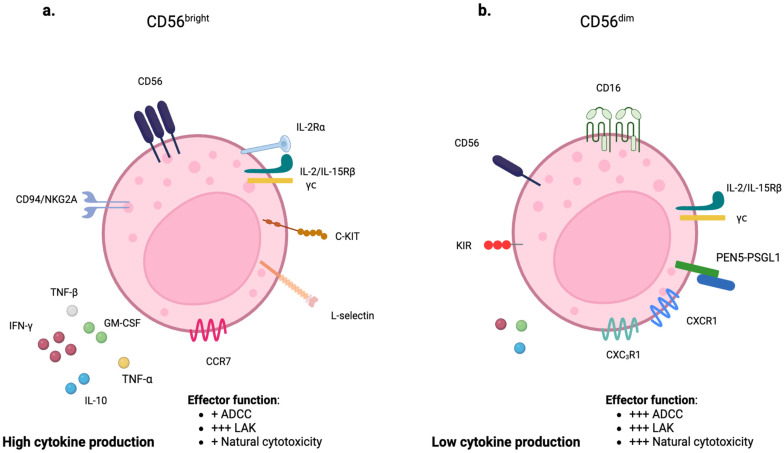
Characteristics and functions of NK-cell subpopulations: CD56bright and CD56dim. (**a**) CD56bright NK cells are characterized by high expression of CD56. They primarily secrete cytokines. (**b**) CD56dim NK cells, on the other hand, are characterized by lower expression of CD56 and higher expression of CD16. They primarily exert cytotoxic activity. ADCC: Antibody-dependent cellular cytotoxicity; LAK: lymphokine-activated killer cell.

Unlike T lymphocytes, NK cells lack CD3 but express the surface molecules CD56 and CD16. CD56 is a transmembrane glycoprotein mediating the interaction between NK and target cells, while CD16 is a low-affinity receptor for the Fc portion of immunoglobulin G (IgG), representing a potent signal to induce Antibody-Dependent Cell-Mediated Cytotoxicity (ADCC). Two subtypes of NK cells can be distinguished based on the rate of CD56 expression: CD56bright and CD56dim NK cells. CD56dim NK cells have high cytotoxic activity and account for approximately 90% of total circulating NK cells. They represent the final stage of NK-cell maturation and play a key role in ADCC due to an increased expression of KIRs, cytotoxic effector proteins (including perforin and granzyme B), and high surface levels of CD16 [7,8]. Conversely, CD56bright includes the remaining 10% and predominates in secondary lymphoid tissues. They are less mature and cytotoxic but secrete cytokines, primarily IFN-γ and TNF-α, in response to interleukin-12 (IL-12) and IL-18 [9] (Figure 3a,b).

## 3. Killer Ig-like Receptors

The KIRs are surface receptors that specifically recognize allelic forms of HLA class I molecules and play a key role in regulating NK cells’ development, tolerance, and activation [1].

The KIR gene cluster consists of 15 genes (KIR2DL1, KIR2DL2, KIR2DL3, KIR2DL4, KIR2DL5A, KIR2DL5B, KIR2DS1, KIR2DS2, KIR2DS3, KIR2DS4, KIR2DS5, KIR3DL1, KIR3DS1, KIR3DL2, KIR3DL3) and two pseudogenes (KIR2DP1, KIR3DP1). It is located on chromosome 19q13.4, within the leukocyte receptor complex.

**Figure 4:**
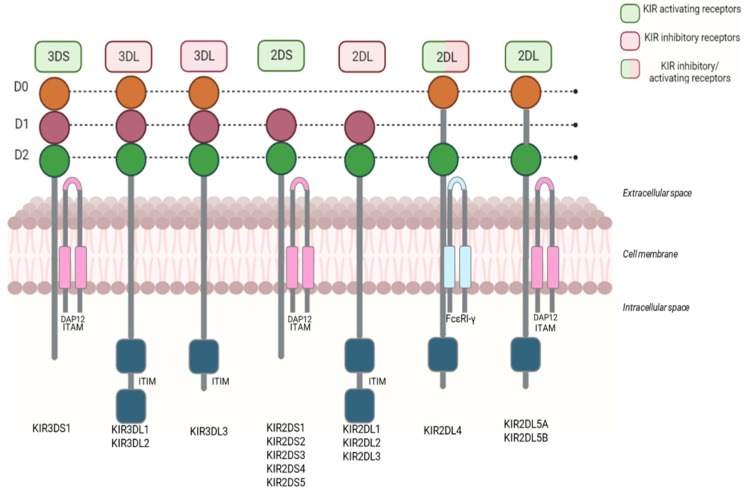
The structure of KIR receptors. KIR receptors consist of three domains: extracellular, transmembrane, and intracellular. The extracellular domain has an Ig-like domain (D0–D2) and the receptor-binding area. The intracellular domain consists of a cytoplasmic tail. Inhibitory KIRs have long cytoplasmic tails with specific motifs (ITIMs). Activating KIRs have short cytoplasmic tails connected to adaptor proteins like DAP12 (ITAM). KIR2DL4 is the only exception, with a long cytoplasmic tail that signals both activation and inhibition.

The structure of KIRs consists of three different domains: (i) extracellular; (ii) transmembrane; (iii) and intracellular (Figure 4). The extracellular domain has two or three domains’ Ig-like, named D1 and D2 in most KIR2D receptors; D0, D1, and D2 in KIR3D receptors; and D0 and D2 in 2DL4 and 2DL5. The transmembrane domain crosses the cell membrane, connecting the extracellular and intracellular domains.

KIRs can be categorized into inhibitors and activators. Inhibitors (KIR3DL1, KIR3DL2, KIR3DL3, KIR2DL1, KIR2DL2, KIR2DL3) regulate NK-cell activity by preventing them from attacking healthy cells. Conversely, activators (KIR3DS1, KIR2DS1-5) stimulate NK-cell action, promoting an immune response against abnormal or infected cells [6].

All inhibitory KIRs have long cytoplasmic tails with immunoreceptor tyrosine-based inhibitory motifs (ITIMs), which recruit tyrosine-phosphatases critical for inhibitory function. Conversely, the activating KIRs, with short cytoplasmic tails, interact with adaptor proteins, such as DAP12 and FcεRI-γ, and contain an immunoreceptor tyrosine-based activation motif (ITAM). KIR2DL4 is the only exception that harbors a unique long-tailed activation of KIR. KIR2DL4 may have both inhibitory and activating action [10].

**Figure 5:**
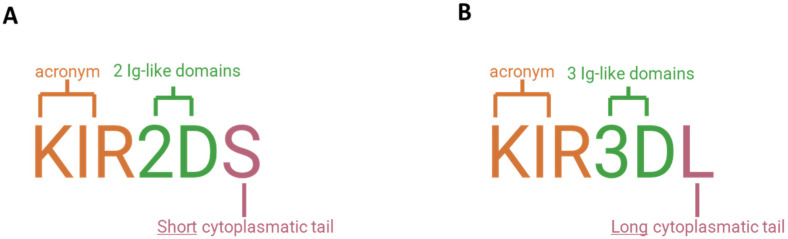
Nomenclature of KIR receptors. (**A**) KIR2DS1, for example, has two extracellular domains (“2D”) and a short intracellular tail (“S”). (**B**) KIR3DL1 has three extracellular domains (“3D”) and a long intracellular tail (“L”).

KIRs have a specific nomenclature. Firstly, based on the number of immunoglobulin-like domains, i.e., two or three, KIRs are denoted with the suffixes 2D or 3D, respectively. The letters L (Long) and S (Short) indicate the length of the intracytoplasmic tail (long or short), corresponding to inhibitory or activating activity. Finally, the number following the letter L or S identifies each protein produced [11] (Figure 5).

**Figure 6:**
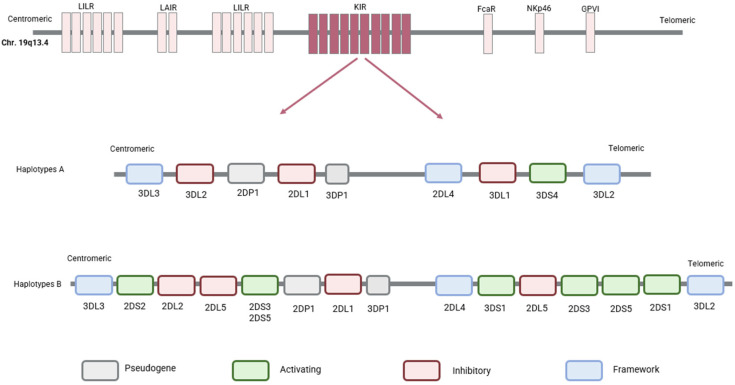
Haplotypes A and B of KIR genes. The KIR gene cluster is located on chromosome 19q13.4. Human NK cells express various combinations of these 17 genes with two common haplotypes: haplotype A and haplotype B. This model identifies the centromeric region, anchored by the framework genes KIR3DL3 and KIR3DP1. Moving towards the telomeric end from KIR3DP1, the framework gene KIR2DL4 marks the telomeric portion, subsequently bounded by the concluding framework gene KIR3DL2.

The KIR genes show a high degree of variation in individual gene content and gene copy number [12]. Human NK cells express various combinations of 17 genes with two common haplotypes: A and B [13]. Each haplotype is divided into two regions, centromeric and telomeric. The centromeric region, anchored by the framework genes KIR3DL3 and KIR3DP1, may include KIR2DS2, KIR2DL2 or KIR2DL3, KIR2DL5, KIR2DS3 or KIR2DS5, KIR2DP1, and KIR2DL1. Moving towards the telomeric end from KIR3DP1, the framework gene KIR2DL4 marks the telomeric portion, subsequently bounded by the concluding framework gene KIR3DL2 (Figure 6). This part can include KIR3DL1, along with either KIR2DS1 or KIR2DS4, or KIR3DS1 with either KIR2DL5 and KIR2DS3 or KIR2DL5 or KIR2DS5, and with either KIR2DS1, KIR1D, or KIR2DS4.

The KIR gene region is highly complex and characterized by high inter-individual variability, contributing to individual diversity in the immune response, and may have implications for disease susceptibility and immunological treatment response [14].

Advancements in gene sequencing technology have revealed a high degree of allelic polymorphism in KIR gene sequencing. Robinson et al. demonstrated the presence of 1532 alleles encoding 668 distinct proteins [15].

### 3.1. Biological Function

In 1990, Ljunggren and Kärre demonstrated that NK cells are activated by the absence of major histocompatibility complex (MHC) class I self-antigens on the target cell in both in vivo and in vitro tumor models [16]. This observation, the “missing self” hypothesis, suggests that the loss of MHC class I on the target cell removes the inhibitory signal, allowing cytotoxic activation of NK cells. Thus, inhibitory receptors recognizing MHC class I contribute to self-tolerance. The expression of self-MHC-I-reactive KIR is also critical for the maturation of functionally responsive NK cells [1].

**Figure 7:**
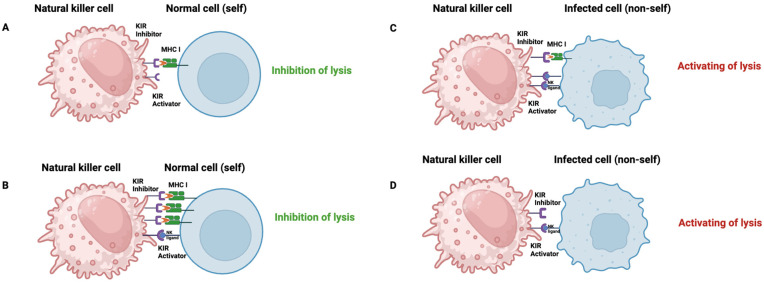
NK-cells activity. It is regulated by a balance of signals from activating and inhibitory receptors: (**A**) In the absence of interaction between the activating receptor and its ligand on the target cell, lysis is inhibited when inhibitory receptors bind to cognate class I MHC molecules on the surface of the target cell or the normal cell (self). (**B**) A predominance of inhibitory receptor–inhibitor interactions with class I MHC results in a net negative signal that prevents NK-cell lysis. (**C**) Activating receptor interactions with ligands on the target cell predominate over weaker MCH class I ligand inhibitory receptor signal, with the net result of NK-cell activation and target cell lysis. This can occur when activating receptors and/or ligands are upregulated, thereby amplifying the net activating signal to overcome the inhibitory signal. (**D**) Lysis occurs when the activating receptor engages its ligand on the target cell in the absence of interactions with the inhibitory receptor and the MHC class I molecule. MCH: Major Histocompatibility Complex; KIR: Killer immunoglobulin-like receptor.

KIR inhibitors mainly protect healthy cells from the cytotoxic effects of NK cells by recognizing MHC class I molecules on the target cell (Figure 7A,B). When autologous HLA is downregulated or absent, as in infected, neoplastic, or allogeneic cells, these receptors cannot convey their inhibitory signals, making the target cell vulnerable to lysis by NK cells (Figure 7C,D).

Activating receptors detect specific stress molecules or signals instructing NK cells to activate and destroy the target cell. If a target cell exhibits numerous stress molecules or signs of infection, the activating receptors are increasingly stimulated, resulting in the activation of NK cells and the subsequent destruction of the target cell. Conversely, if the target cells express many inhibitory molecules, the inhibitory signal predominates, thereby preventing NK-cell action against those specific cells.

### 3.2. KIRs and HLA Interaction

**Figure 8:**
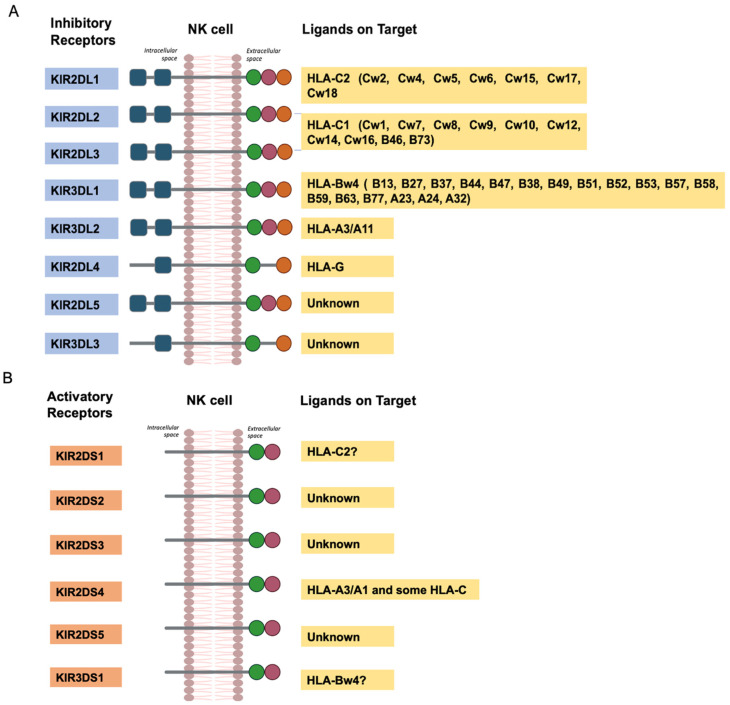
The interaction of KIR with HLA ligands. (**A**), inhibitory receptors. (**B**), activatory receptors.

As shown in Figure 8, the interaction between KIRs and specific HLA class I molecules regulates the functions of cytotoxic cells in the immune system [17].

However, since KIR genes and HLA genes are located on different chromosomes, an individual may possess a KIR gene without having its corresponding HLA ligand [18].

In addition, single nucleotide polymorphisms (SNPs) can alter the receptor’s specificity for HLA class I ligands and reduce receptors’ surface expression.

The interaction between KIR and HLA represents a critical checkpoint in immune surveillance, influencing the balance between immune activation and tolerance. Functionally, KIR-HLA interactions regulate NK-cell education, cytotoxicity, and cytokine production, thereby shaping immune responses in health and disease.

In the context of infection, certain KIR-HLA combinations enhance NK-cell responsiveness, contributing to effective viral clearance. For instance, the presence of activating KIRs such as KIR3DS1 in combination with HLA-Bw4 has been associated with delayed progression of HIV, highlighting their protective role. Conversely, inhibitory KIR-HLA interactions may dampen NK-cell responses, allowing persistent infections or contributing to immune evasion by pathogens.

In cancer, the loss or downregulation of HLA class I molecules, a common mechanism by which tumors escape cytotoxic T lymphocytes, can trigger NK-cell activation, particularly when inhibitory KIRs fail to engage their ligands. The diversity of KIR and HLA genotypes among individuals also influences the efficacy of NK-cell-mediated immunosurveillance, and certain KIR-HLA mismatches are being exploited in hematopoietic stem cell transplantation to improve graft-versus-leukemia effects.

KIR-HLA dynamics also shape autoimmune and inflammatory diseases. For example, specific inhibitory KIR-HLA combinations have been linked to an increased risk of autoimmune conditions like rheumatoid arthritis and psoriasis, potentially due to dysregulated NK-cell inhibition and increased tissue damage.

Overall, the functional interplay between KIRs and HLAs is central to immune regulation, and its genetic diversity contributes to interindividual variability in disease susceptibility, progression, and treatment outcomes. Understanding these mechanisms offers valuable insights into developing personalized therapeutic strategies, including NK-cell-based immunotherapies and transplant compatibility assessments [14].

## 4. KIR Genes in Autoimmune Diseases

**Figure 9:**
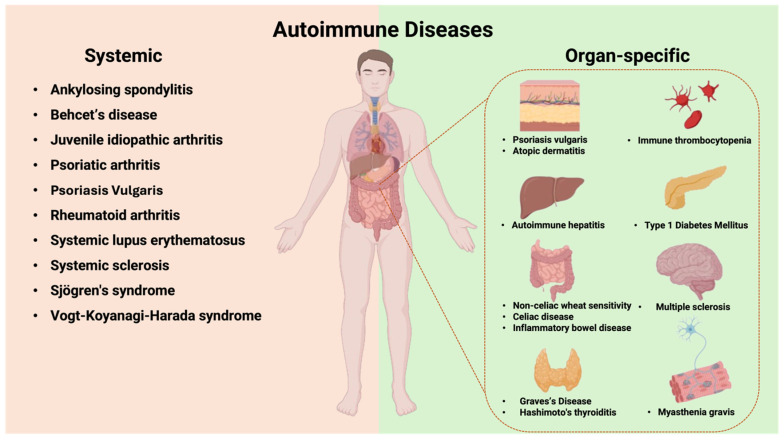
Systemic and organ-specific autoimmune diseases: classification based on autoantibody localization.

Autoimmune diseases are a group of chronic disorders characterized by aberrant adaptive immune responses to self-antigens [19]. Autoimmune diseases are classified as either organ-specific or systemic based on the localization of autoantibodies (Figure 9). In organ-specific autoimmune diseases, the immune responses target antigens present only in specific tissues or organs, with end-organ damage mediated by antibodies and/or T cells. In systemic autoimmune diseases, the autoimmune response targets self-antigens expressed in multiple organs and tissues, with end-organ injury typically mediated by autoantibodies and, less commonly, T cells. The development of autoimmunity and its progression to autoimmune disease occurs on a continuum, involving a complex interplay between genetic and environmental factors over time. Genetic risk variants and epigenetic alterations predispose individuals to a loss of immune tolerance, leading to the subsequent development of autoantibodies.

KIR genes are critical in modulating immune responses, and their association with autoimmune diseases has been extensively explored [20]. The delicate balance between activating and inhibitory signals mediated by KIRs is essential for maintaining immune homeostasis (Table 1). For instance, specific activating KIRs can lead to a loss of immune tolerance, potentially triggering the onset of autoimmune disorders. Conversely, inhibitory KIRs may offer protection by suppressing excessive immune activation. The independent inheritance of KIR and HLA genes generates different possible combinations with distinct implications for autoimmune disease susceptibility. Investigating KIR genes and their role in autoimmune disease susceptibility remains a central focus in immunogenetics, offering potential breakthroughs in diagnosing, preventing, and treating autoimmune disorders.

### 4.1. Ankylosing Spondylitis

Ankylosing spondylitis (AS) is a chronic inflammatory disease primarily affecting the spine and sacroiliac joints, leading to pain and progressive spinal stiffness. The exact pathogenesis of AS is not fully understood, but it is known to have a strong genetic component. The association between HLA-B27 and AS remains the most robust link identified between HLA and the disease [21,22].

Some authors have explored the association between KIRs and AS, suggesting that KIR-HLA combinations can influence disease susceptibility and progression. Individuals with certain activating KIRs and the HLA-B27 allele may have a higher risk of developing AS. Lopez-Larrea et al. observed that the presence of KIR3DS1 or KIR3DL1 in combination with HLA-B*27s/HLA-B HLABw4-I80 genotypes modulate AS onset in the Caucasian population [23]. Particularly, KIR genes may modulate NK-cell cytokine secretion (e.g., downregulation of interferon γ) and adhesion functions through its interaction with HLA-B27.

Similarly, Wang et al. and Kuijpers et al. reported that KIR3DS1, in addition to HLA-B27, may play a significant and independent role in AS pathogenesis in the Chinese population [24,25]. However, data from the literature are still inconclusive and incongruous. Fan et al. reported that KIR2DS4 and KIR3DS1 might be potential risk factors for AS. Of note, they observed a positive association between KIR2DS4 and susceptibility to AS in Asians, but not in Caucasians, and a positive association between KIR3DL1, KIR3DS1, and susceptibility to AS in Caucasians, but not in Asians [26]. In addition, a recent meta-analysis, including 1770 cases and 2907 healthy subjects, indicates that KIR2DS1, KIR2DS5, and KIR3DS1 polymorphisms increased AS risk, while KIR2DL2 and KIR2DS2 polymorphisms are associated with reduced AS susceptibility [27]. Further research is mandatory to confirm the initial observations.

### 4.2. Atopic Dermatitis

Atopic dermatitis (AD) is a chronic inflammatory skin disease characterized by dry skin, intense itching, and rash. It is one of the most common dermatological disorders, especially in children, and its pathogenesis involves a complex interplay of genetic, environmental, and immunological factors. Although the role of NK cells in AD development has recently been postulated [28,29], the involvement of KIR genes in the diseases is still poorly investigated. Niepiekło-Miniewska et al., for the first time, reported a protective role of KIR2DS1 in AD, with the frequency of KIR2DS1 in AD patients smaller than controls [30]. Conversely, a recent case-control study showed that KIR2DS1, KIR2DL5, and KIR2DS5 increased the risk of AD [31]. In addition, authors also reported that KIR alleles, including KIR2DS4*001:01 and KIR2DL4*001:02, in the presence of HLA C*04:01 are associated with AD severity in children over time [32]. The direct mechanism for KIR genes’ involvement in AD onset is still unknown. However, some authors suppose that the increased AD risk is due to the presence of these genes in the setting of less-educated NK cells, leading to decreased circulating numbers of NK cells and diminished NK-cell function.

### 4.3. Autoimmune Hepatitis

Autoimmune hepatitis (AIH) is an autoimmune liver disease caused by a loss of immunological tolerance to autologous liver tissue. This leads to hepatocellular inflammation, characterized by elevated levels of circulating autoantibodies, hypergammaglobulinemia, and fluctuating increases in serum transaminases and IgG levels.

Different disease subtypes are categorized based on circulating autoantibodies. Type 1 AIH is characterized by the presence of antinuclear antibodies (ANAs), anti-smooth muscle antibodies (SMAs), and anti-soluble liver antigen/liver-pancreas (anti-SLA/LP). Type II AIH represents 10% of all cases of AIH and is characterized by positivity for anti-liver kidney microsome 1 antibody (LKM1) and anti-liver-cytosol type 1 (anti-LC1) [33].

Increasing evidence confirms that greater or lesser susceptibility to disease is associated with specific HLA class I (A*01, B*08) and class II alleles (DRB1*03, −04, −07 or −13), depending on geographic regions [34,35,36].

The role of KIR in AIH has not been fully clarified. Littera et al. reported that activating the KIR2DS1 gene is more frequent in type I AIH patients than in healthy controls [37]. In addition, AIH patients have low frequencies of KIR2DL3 and KIR3DL1, along with their ligands (HLA-C1 and HLA-Bw4).

The same year, Podhorzer et al. showed the association between pediatric AIH and HLA-DRB1*1301. They identified a high frequency of KIR2DS4 and a reduced frequency of the inhibitory KIR2DL2 gene and HLA-C1 ligands, indicating altered inhibitory mechanisms [38]. These findings suggest a potential role for KIR genes in influencing susceptibility or protection against AIH.

Further studies have identified a significant association between specific KIR genes, such as KIR3DL1 and KIR2DL1, and their HLA ligands, including HLA-B-Bw4-80 Ile, HLA-C2, and HLA-B Bw4-80Thr in Japanese patients with type I AIH [39]. Specifically, KIR3DL1/HLA-B Bw4-80Ile are strongly linked to the disease and act as an independent susceptibility gene, along with the HLA-DRB1 haplotype. In AIH patients, lower frequencies of KIR2DL1/HLA-C2 and KIR3DL1/HLA-B Bw4-80Thr suggest a protective role for these KIR-HLA pairs. Additionally, KIR3DL1/HLA-B Bw4 and KIR3DL1/HLA-B Bw4-80Ile are protective against liver decompensation and related deaths, while the absence of KIR3DL1/HLA-B Bw4 and the presence of cirrhosis at diagnosis are associated with disease progression. This finding highlights the importance of the interaction between KIR receptors and HLA ligands in the risk of developing AIH.

The apparent duality between increased activating KIRs (e.g., KIR2DS1, KIR2DS4) and decreased inhibitory KIRs (e.g., KIR2DL2, KIR2DL3, KIR3DL1) in AIH reflects the underlying complexity of immune dysregulation in this disease. Both enhanced immune activation and insufficient inhibitory signaling likely contribute to AIH pathogenesis in a complementary rather than mutually exclusive manner. The overrepresentation of activating KIRs may promote heightened NK- or T-cell activity against hepatic tissue, while the underrepresentation of inhibitory KIRs may result in a reduced threshold for immune cell activation and a breakdown of tolerance. This imbalance, a shift toward activation without an adequate inhibitory counterbalance, can create a permissive environment for autoimmune attacks on liver tissue. As such, AIH appears to be driven by both excessive immune activation and a failure of inhibitory control, with variations depending on genetic background and disease subtype.

In conclusion, the emerging evidence enhances the complex interplay between KIR genes and HLA ligands in AIH. However, further studies are necessary to elucidate the mechanisms by which these genetic factors influence disease susceptibility and progression.

### 4.4. Behçet’s Disease

Behçet’s disease (BD) is a chronic, multisystem inflammatory disorder characterized by recurrent oral and genital ulcers, uveitis, and skin lesions. This condition can also involve other organs, such as joints, the vascular system, and the central nervous system. The etiopathogenesis of BD is unclear, and genetic studies have focused on identifying associations between specific genes and the risk of BD, including KIR genes and HLA variants [40,41]. Notably, the HLA-B51 allele is the genetic factor most associated with the development and progression of BD.

The role of KIR genes in BD has been explored in several studies. Firstly, Middleton et al. observed no significant association between KIR genes and BD [42]. Similarly, other authors reported no association between KIR genes and BD susceptibility [43,44].

Conversely, Erer et al. reported, for the first time, a potential role for activating KIR3DS1 alleles in the ocular manifestations of BD, independent of HLA-B51, the most substantial susceptibility factor for BD. These results indicate that the pathogenic mechanism of KIR3DS1 in ocular involvement does not rely on HLA-B51 [45].

In addition, Castano-Nunez et al. observed a significant association between KIR3DL1*004 and BD susceptibility. The authors suggest that this allele has a protective role in the development of the disease and that its effect is independent of HLA-B51 [46].

Overall, studies highlight that the role of KIR genes in BD pathogenesis is unclear and requires further investigation to understand their specific contributions.

### 4.5. Celiac Disease

Celiac disease (CD) is a chronic autoimmune disorder affecting the small intestine, triggered by the ingestion of gluten in individuals genetically predisposed. This condition is characterized by an inappropriate immune response to gluten, leading to inflammation and damage to the intestinal villi. CD symptoms range from gastrointestinal dysfunctions such as diarrhea, bloating, and abdominal pain to extra-intestinal manifestations like anemia, osteoporosis, and neurological alterations.

The most important genes for CD predisposition are the leukocyte histocompatibility antigen genes HLA-DQ2 encoded by (HLA-DQA1*05-DQB1*02) and HLA-DQ8 encoded by (DQA1*03-DQB1*0302) on chromosome 6p21. The HLA-DQ2 and HLA-DQ8 haplotypes are expressed in 90% and 5% of CD patients, respectively [47].

In CD pathogenesis, gluten peptides, particularly gliadin, are deamidated by the enzyme tissue transglutaminase (tTG). These altered peptides are subsequently presented to CD4+ T cells by antigen-presenting cells through HLA-DQ2 or HLA-DQ8 molecules. This immune activation triggers an inflammatory response, resulting in damage to the intestinal mucosa, villous atrophy, and impaired nutrient absorption [48]

The gene cluster KIR is a potential candidate locus for CD susceptibility due to its involvement in the innate immune response and location on chromosome 19q13.4, a region linked to CD susceptibility.

Moodie et al. are the first to propose a potential role for KIRs in CD pathogenesis. However, the authors failed to find any association between CD and the common KIR genotype and haplotype [49].

Further, Santin et al. reported the involvement of KIR2DL5B in CD onset, although the mechanisms by which the gene confers susceptibility to the disease remain unclear [50].

In 2011, Fernandez-Jimenez et al. highlighted a significant expression of the KIR3DL1 gene in CD patients compared to healthy individuals [51]. Caggiari et al. found higher frequencies of KIR2DL2/HLA-C1, KIR2DS2, KIR2DL5B, and KIR2DS3 in CD patients experiencing complications, such as cancer [52].

Other authors revealed that patients with coexisting Type 1 Diabetes Mellitus (T1DM) and CD frequently exhibit the presence of HLA-C07, which is associated with the KIR ligand C1 [53]. Akar et al., in a case-control study, showed that activating KIRs, specifically KIR2DS5 and KIR3DS1, along with the class I HLA-C1 ligand, could be associated with an increased risk of CD [54]. Additionally, the authors found that specific combinations of KIRs and HLA class I ligands could either increase the risk or protect against the development of CD.

Overall, these data suggest the involvement of KIR polymorphisms in CD pathogenesis, highlighting that innate immune responses may also play a crucial role in disease susceptibility and progression.

### 4.6. Graves’ Disease

Graves’ disease (GD) is an autoimmune disorder that primarily affects the thyroid gland. It is characterized by the production of autoantibodies that activate the thyroid-stimulating hormone (TSH) receptors on thyroid cells [55]. These antibodies induce follicular hypertrophy and hyperplasia, resulting in thyroid enlargement and increased thyroid hormone production, ultimately causing thyrotoxicosis. Symptoms of GD include weight loss, increased heart rate, heat intolerance, and anxiety.

Genetic predisposition likely contributes to the breakdown of self-tolerance, triggering the autoimmune response. In addition, abnormalities in NK-cell counts, activity, and cytokine secretion have been observed in GD patients [56].

In 2009, Zhang et al. reported the first association between KIR gene polymorphisms and GD onset. The authors observed that genotypes without activating KIR genes, defined by KIR2DS2-, KIR2DL2-, KIR2DL3+, KIR2DL1+, KIR3DL1+, KIR3DS1-, KIR2DL5-, KIR2DS3-, KIR2DS5-, KIR2DS1-, and KIR2DS4-, have a higher frequency in patients than healthy controls [57]. Conversely, Ashouri et al. and Dastmalchi et al. failed to find significant associations between KIR gene variants and GD [58,59], suggesting that the contribution of KIR gene polymorphism to NK dysfunction and other autoimmune abnormalities observed in GD appears to be minimal.

Recently, a significant association between KIRs and GD has been reported. Specifically, low expression of KIR2DL1/HLA-C2 in GD patients may act as a protective factor for the disease [60].

In summary, HLA alleles are strongly associated with GD risk, while the role of KIR gene variants remains unclear and requires further studies to clarify their impact.

### 4.7. Hashimoto’s Thyroiditis

Hashimoto’s thyroiditis (HT) is a chronic autoimmune thyroid disease characterized by an increased thyroid volume, lymphocyte infiltration, and autoantibodies against thyroid antigens. The pathogenesis of HT involves an interplay of environmental triggers, genetic factors, such as HLA variants, and immune dysregulation. Autoimmune responses against thyroid antigens, such as thyroid peroxidase (TPO) and thyroglobulin, lead to chronic inflammation and destruction of thyroid tissue. This results in a gradual decline in thyroid hormone production, ultimately leading to hypothyroidism [61].

Recent studies have investigated the role of KIR genes in HT predisposition. Ashouri et al. found no significant differences in KIR gene variants between patients and healthy controls [62]. Successively, a significantly increased frequency of the KIR2DS2/HLA-C1 combination has been observed in HT patients compared to controls, while the frequency of the KIR2DS2-/KIR2DL2+/KIR2DL3+/HLA-C1 combination was significantly reduced [63].

In conclusion, HT has a multifactorial etiology where genetic susceptibility, particularly HLA and potentially KIR genes, interacts with environmental triggers to initiate and perpetuate autoimmune thyroid inflammation. Further research into the specific mechanism by which KIR genes influence HT susceptibility could provide valuable insights into disease pathogenesis.

### 4.8. Immune Thrombocytopenia

Autoimmune thrombocytopenia, also known as immune thrombocytopenic purpura (ITP), is a hematologic disorder characterized by a low platelet count resulting from the immune system attacking and destroying platelets. This condition occurs when the immune system targets platelet surface antigens with autoantibodies, leading to their clearance by phagocytes in the spleen and liver, as well as potentially inhibiting platelet production by bone marrow megakaryocytes. The exact triggers for autoantibody production in ITP remain unclear but may involve viral infections, medications, and other autoimmune conditions as contributing factors. ITP can manifest with easy or excessive bruising, bleeding, and petechiae (small red or purple spots on the skin).

Olsson et al. first explored the association between KIR genes and ITP, showing that the inhibitory KIR2DL3, KIR3DL2, and KIR3DL1 are upregulated in T cells of patients in remission compared to those with active ITP [64]. Subsequently, other studies revealed that the KIR2 genotype is overexpressed in patients with ITP. Specifically, they observed that the combination of KIR2DS2/KIR2DL2 was more prevalent in ITP patients than in controls [65,66]. In a prospective case-control study, Seymour LA et al. further investigated the influence of KIR variants in adult patients with chronic and relapsed ITP. They identified that the presence of KIR2DS5 conferred a protective influence against ITP independently of other KIR genes and HLA-C allotypes. Conversely, KIR2DS2 and KIR2DS3 were associated with an increased risk of developing ITP. These findings underscore the intricate role of KIR genes in modulating immune responses and suggest potential implications for the understanding and managing of adult ITP [67].

Studies on KIR genes provide valuable insights into the immunogenetic factors influencing susceptibility to ITP and could lead to target therapeutic approaches in the future.

### 4.9. Inflammatory Bowel Disease: Crohn’s Disease and Ulcerative Colitis

Inflammatory bowel disease (IBD) includes chronic inflammatory conditions of the gastrointestinal tract, primarily Crohn’s disease (CrD) and ulcerative colitis (UC). Both conditions are characterized by active inflammation and remission periods, leading to symptoms such as abdominal pain, diarrhea, weight loss, and fatigue. The etiology of IBD is multifactorial, involving genetic predisposition, environmental factors, gut microbiota, and immune system dysregulation.

Research investigating KIR genes and their interactions with HLA alleles in IBD has revealed significant insights. Indeed, the role of KIR-HLA interactions, particularly in NK cells and CD8+ T cells, is increasingly recognized in contributing to disease pathogenesis. Mechanistic pathways in IBD involve loss of inhibitory signaling, leading to increased inflammation, inappropriate activation of CD8+ T Cells, and impaired NK-cell education. Indeed, NK cells are “educated” during development via interactions between inhibitory KIRs and self-HLA. In individuals with KIR-HLA mismatches, NK cells may become hyporesponsive or improperly tuned: either too aggressive (if over-activated) or unable to eliminate dysregulated immune cells (if hyporesponsive). In IBD, this can contribute to failure to clear activated T cells or prolonged intestinal inflammation. Finally, in IBD, tissue-resident NK and CD8+ T cells expand in the lamina propria. In some patients, KIR+ CD8+ T cells, especially those expressing KIR2DL1 or KIR3DL1, may become chronically activated, contributing to fibrosis, mucosal damage, and steroid resistance.

Initial studies suggested a potential role of KIR2DS2 and KIR2DL2 in UC pathogenesis. Subsequent analyses underscored a protective association of KIR2DL3 with HLA-Cw1 [68]. In the Japanese population, the HLA-Bw4 allele was significantly associated with both UC and CrD, while KIR2DS3 was identified as a risk factor for UC, and the KIR3DL1-HLA-Bw4 combination showed associations with both UC and CD [69].

Regarding CrD, some studies have highlighted the prevalence of KIR2DL3/HLA-C1, indicative of a weak inhibitory response potentially contributing to disease susceptibility. Conversely, KIR2DL2 and KIR2DS2 were associated with a reduced risk of CrD [70].

Meta-analyses further supported these findings, identifying specific KIR genes, including KIR2DS1 and KIR2DL5, with increased UC risk, and KIR2DS3 with decreased CrD risk [71]. These findings suggest that KIR genes may influence the balance between activating and inhibitory immune responses in the context of IBD [72,73]. Further large-scale studies are warranted to validate these associations and elucidate the underlying mechanisms, paving the way for future advancements in IBD treatment.

### 4.10. Juvenile Idiopathic Arthritis

Juvenile Idiopathic Arthritis (JIA) is a heterogeneous group of chronic rheumatic diseases affecting children (under 16 years of age), characterized by persistent joint inflammation lasting more than six weeks. JIA encompasses several subtypes, including oligoarthritis, polyarthritis, systemic JIA, enthesitis-related arthritis (ERA), and others. Studies have provided insights into the role of KIR genes in JIA. Zhou et al. found no association between KIR genes and JIA, except for the KIR2DS4 gene, significantly lower expressed in patients with systemic JIA [74]. A subsequent study investigated the role of KIR genes in patients with ERA, a subtype of JIA. These patients showed increased IL-17 production by NK cells. Additionally, patients had higher KIR3DL1/KIR23DL2 expression and HLA-B27 positivity, underscoring the potential involvement of NK cells in the pathogenesis of the disease through abnormal interactions involving HLAB27 and KIR3DL1/KIR3DL2 [75].

Different subtypes of JIA may exhibit distinct immunogenetic profiles, such as the involvement of KIR2DS4 in systemic JIA and KIR3DL1/KIR3DL2 in ERA, underscoring the variability and complexity of autoimmune mechanisms in these diseases. Further elucidation of these genetic and immunological pathways could lead to targeted therapies improving the management of children affected by JIA.

### 4.11. Myasthenia Gravis

Myasthenia gravis (MG) is an autoimmune neuromuscular disorder characterized by weakness and rapid fatigue of the voluntary muscles. This condition results from an immune-mediated attack on acetylcholine receptors (AChRs) at the neuromuscular junction, impairing communication between nerves and muscles. The hallmark symptoms of MG include ptosis (drooping eyelids); diplopia (double vision); and generalized muscle weakness, which often worsens with activity and improves with rest.

The pathogenesis of MG is primarily driven by the activation of autoreactive B cells producing autoantibodies. The predominant autoantibodies belong to the IgG1 and IgG3 classes, targeting AChR, which are detected in approximately 85% of generalized MG patients and 50% of ocular MG patients. Other autoantibodies, such as muscle-specific tyrosine kinase (MuSK), can target essential proteins at the neuromuscular junction. Beyond the roles of B and T cells in MG, NK cells also contribute to its development. NK cells primarily function to defend the body against cancerous cells or invading pathogens, but their involvement in MG adds another layer of complexity to the disease’s immune dysregulation. To date, only one study explored the role of KIR in MG, showing no significant difference in the frequency of KIR genes and inhibitory KIR genotypes between controls and patients [76].

### 4.12. Multiple Sclerosis

Multiple Sclerosis (MS) is an autoimmune disease of the central nervous system characterized by chronic inflammation that damages the myelin and the nerve fibers.

Its multifactorial etiology involves complex interactions between genetic susceptibility, environmental factors, and immune dysregulation. One of the hallmarks of MS is the infiltration of autoreactive immune cells, particularly CD4+ T-helper cells (such as Th1 and Th17 subsets), into the CNS, where they initiate and sustain inflammation, leading to demyelination and axonal damage [77,78]. Also NK cells play a crucial role, and their activity is regulated by interactions between KIR receptors and HLA class I ligands on target cells [79].

Juan A. García-León and colleagues examined the role of the KIR-HLA system in MS in a cohort of Spanish patients, confirming the protective role of the HLA-Bw4 motif against MS. Additionally, potential associations were identified between the KIR3DS1 and KIR2DL5 genes and susceptibility to MS, while the KIR2DL1 and KIR2DS5 genes were associated with increased disease severity [80]. Similarly, in a study involving a northern Portuguese population, Bettencourt et al. found a negative association between KIR2DS1 and MS, suggesting a protective role. Comparable findings were reported in studies involving Norwegian and Italian populations [81,82,83]. In a separate study examining patients with clinically isolated syndrome and confirmed MS, Jelcić et al. observed reduced frequencies of the KIR2DL3 gene among patients. Additionally, they noted a higher prevalence of individuals lacking KIR2DL3 but possessing two copies of KIR2DL2/KIR2DS2 in both cohorts [84].

Hollenbach et al. conducted a study in an African American cohort that reinforced the protective role of the KIR3DL1 ligand HLA-Bw4 against MS, highlighting its functional significance across diverse ancestral backgrounds [85]. Around the same time, Shahsavar et al. published a meta-analysis supporting the protective effect of KIR2DS1 against MS, potentially through the activation of NK cells, which suppress autoreactive T cells [86].

A recent study examined HLA ligand/KIR genotype combinations in DRB1*15:01-negative individuals, identifying a significant protective association between HLA-Bw4 and KIR2DL2/KIR2DL3. This association appeared potentially stronger than that with KIR3DL1, though further validation in larger cohorts is required [87].

In conclusion, KIR genes play critical roles in MS susceptibility and severity by interacting with HLA class I ligands. These genetic associations enhance our understanding of MS pathogenesis and offer potential targets for therapeutic interventions to modulate immune responses mediated by KIR receptors.

### 4.13. Non-Celiac Wheat Sensitivity

Non-Celiac Wheat Sensitivity (NCWS) is a condition characterized by adverse reactions to wheat and gluten-containing foods in individuals who do not have CD or wheat allergy. NCWS lacks the typical markers of CD but shares some symptomatic similarities. While CD primarily involves the adaptive immune system, NCWS is characterized by prominent activation of the innate immune response. This distinction is essential to understanding the role of innate immune components, including KIR genes, in the development and progression of NCWS [88].

To date, only one study has explored the potential role of KIR genes in NCWS susceptibility. The authors found that NCWS patients exhibited distinct patterns of KIR gene expression compared to CD patients and healthy individuals. In particular, KIR genes like KIR2DL5, KIR2DS4, and KIR2DS5 were negatively associated with NCWS susceptibility, suggesting their potential protective role [89].

In conclusion, while the exact mechanisms underlying NCWS are not fully elucidated, the distinct immune activation pattern involving innate immunity and the preliminary findings on KIR genes suggest a complex interplay of genetic and environmental factors in the manifestation of NCWS. Further research is essential to validate these findings and explore additional genetic markers and immune pathways that may contribute to the understanding and managing of NCWS.

### 4.14. Psoriatic Arthritis

Psoriatic arthritis (PsA) is an autoimmune disease that affects the joints and skin, often occurring in individuals with a pre-existing condition of psoriasis. Genetic studies, including those investigating KIR genes, have shed light on the genetic susceptibility to psoriatic arthritis (PsA). Genes such as KIR2DL2, KIR2DS1, KIR2DS2, and KIR2DS3 have been positively linked to an increased predisposition to PsA in Caucasian populations. Follow-up studies have reinforced these findings, confirming the positive associations of activating KIR genes (KIR2DS1 and KIR2DS2) as well as the inhibitory gene KIR2DL2 [90,91,92,93,94].

A meta-analysis confirms positive associations between KIR2DL1, KIR2DS1, KIR2DS2, and KIR2DS3 genes and susceptibility to PsA. Ethnicity-specific analyses showed positive associations in Caucasians, while analysis for the Asian population was not conducted due to limited data availability [95].

In conclusion, while genetic predisposition involving KIR genes contributes to the susceptibility to PsA, the exact mechanisms by which these genes influence disease onset and progression require further elucidation.

### 4.15. Psoriasis Vulgaris

Psoriasis vulgaris (PsV) is a chronic autoimmune skin disease characterized by red, scaly patches on the skin, which can appear on the elbows, knees, scalp, and trunk. The disease is due to an overactive immune response leading to the rapid proliferation of skin cells. T cells play a significant role in this process, mainly by releasing pro-inflammatory cytokines, such as TNF-alpha, IL-17, and IL-23. Genetic studies have identified 60 genes involved in PsV, including KIR genes and MHC-1 alleles, which affect the immune response and susceptibility to the disease [96].

Studies on Caucasian, Asian, and mixed populations have shown susceptibility links between psoriasis or some clinical variations and KIR genes or KIR/HLA composite genotypes [91,97,98,99,100,101]. A recent meta-analysis revealed that the KIR2DS1 gene is associated with an increased risk of PsV, whereas KIR2DS4 and KIR3DL1 may offer protective effects. Among Caucasians, KIR2DS1 is the only gene linked to PsV, and it is considered a potential risk factor. In contrast, in Asian populations, KIR2DL1, KIR2DS4, and KIR3DL1 are suggested to act as protective factors against PsV, while KIR2DS5 and KIR3DS1 may increase susceptibility to the condition [102].

### 4.16. Rheumatoid Arthritis

Rheumatoid arthritis (RA) is a chronic autoimmune disease that affects the joints, causing inflammation, pain, swelling, and potential joint damage. This systemic condition occurs when the immune system attacks the synovial membranes, the tissues lining the joints.

Research into the genetic factors contributing to RA has highlighted the intriguing role of KIR genes. The first study exploring the relationship between KIR genes and RA identified KIR2DS2 as being implicated in developing vasculitis-associated RA [103]. Later studies focusing on the Lur population in Iran revealed that KIR2DL3 and KIR2DL5A play protective roles against RA, while the full-length variant of KIR2DS4 was associated with an increased risk of developing the disease. Furthermore, KIR2DL2 and KIR2DS2 were linked to heightened RA susceptibility, whereas KIR2DL3 consistently showed protective effects [104].

Li X et al. conducted a meta-analysis revealing geographic and genetic variations in KIR gene associations with RA. They noted positive associations of KIR2DL1 and KIR2DS1 in East Asians, while KIR2DL3 showed a negative association [105]. Aghaei H et al. further consolidated these findings, identifying KIR2DL3, KIR2DL5, KIR2DS5, and KIR3DL3 as significantly negatively associated with RA development [106].

Regarding treatment response, the relationship between KIR genes and RA has also been assessed in the response to drugs in RA patients. It was observed that RA patients with KIR2DS2 and KIR2DL2 polymorphisms have a better response to methotrexate [107]. Ramírez S. and colleagues explored the role of KIR genes in relation to anti-cyclic citrullinated peptide (anti-CCP) serodiagnosis in RA patients from western Mexico, revealing associations of KIR2DL2 with anti-CCP positivity and KIR2DL3 with protection against RA, particularly in anti-CCP negative patients [108]. Recent studies in the Lur population of Iran confirmed the protective effects of KIR2DL3 and KIR2DL5A against RA, contrasting with the increased risk associated with KIR2DS4full [109].

These findings underscore the complex interplay of KIR genes in RA pathogenesis, susceptibility, and treatment response across different populations. Future research should aim for larger, more robust studies to elucidate these genetic mechanisms further and potentially inform personalized approaches to RA management.

### 4.17. Sjögren’s Syndrome

Sjögren’s syndrome (SS) is a chronic autoimmune disorder characterized by inflammation that primarily affects the salivary and lacrimal glands, resulting in mucosal dryness, especially in the mouth and eyes. In SS, the pathogenesis involves the immune system attacking the exocrine glands, leading to lymphocytic infiltration and subsequent glandular dysfunction. The genetic susceptibility to SS is known, with a high frequency of cases carrying the MHC antigens HLA-A1, HLA-B8, and HLA-DR3 [110].

The first study linking KIR genes and SS found that the KIR2DS2+/KIR2DL2- combination was more common in SS patients than controls. Additionally, SS patients carrying both the KIR2DS2 gene and the corresponding HLA-C1 ligand, without KIR2DL2, were significantly more prevalent than in the control group [111].

In conclusion, the association between KIR genes and SS highlights the complex interplay of genetic factors in autoimmune diseases. Further research is needed to elucidate the specific roles of KIR genes in SS pathogenesis and their implications for clinical practice.

### 4.18. Systemic Lupus Erythematosus

Systemic Lupus Erythematosus (SLE) is a chronic autoimmune disease characterized by the production of autoantibodies directed against various organs and tissues, resulting in a wide range of clinical manifestations. Genetic predisposition plays a significant role in SLE susceptibility. Numerous studies have reported gene alterations in immune regulation and signaling pathways, highlighting their potential roles in disease development and progression. Of note, KIR genes have garnered attention for their role in modulating NK- and T-cell responses, with activating KIR genes like KIR2DS1 and KIR2DS2 associated with increased susceptibility to SLE by promoting NK-cell activation and pro-inflammatory cytokine production [112,113]. Conversely, inhibitory KIR genes such as KIR2DL5 may confer protection against SLE by tempering immune responses, although this protection may come at the cost of increased susceptibility to infections [114].

Further investigations have underscored the association between activating KIR genes and SLE, particularly, KIR2DS1 and KIR2DL2. These genes correlate with aberrant T-cell activation and heightened cytokine production, such as IL-1, IL-6, and TNF-α, potentially contributing to disease pathogenesis [115,116]. In contrast, Akhtari et al. found no significant differences in the frequency of activating KIR genes between SLE patients and controls in an Iranian population However, they highlighted interactions between specific KIR and HLA alleles influencing clinical manifestations of SLE, such as hematological and renal disorders [117]. Tozkır et al. explored KIR gene associations in autoimmune connective tissue diseases, including SLE. They found significant associations of KIR2DS2 with disease susceptibility, particularly in the absence of inhibitory KIR genes like KIR2DL2 [118]. Liang et al., in a meta-analysis, identified KIR2DL3 and KIR3DL1 as prevalent genes associated with SLE, suggesting variations across populations in KIR gene variants [119].

Furthermore, Gambino et al. highlighted a correlation between the inhibitory KIR2DL5B gene, activating KIR2DS2 genes, and specific HLA alleles (HLA-A-Bw4 and HLA-C1) with SLE. They noted that HLA-C1 was more prevalent in SLE patients than controls, implying its potential role in disease pathogenesis. Specific KIR and HLA combinations, such as KIR2DL2/HLA-C1, KIR2DL3/HLA-C1, and KIR2DS2/HLA-C1, were also implicated in SLE, suggesting their critical involvement in disease mechanisms [120].

Moreover, Segerberg et al. investigated autoantibodies targeting KIRs in SLE patients, observing associations with higher disease activity, elevated IFN-α levels, and increased nephritis risk. They proposed that these autoantibodies could influence NK-cell function in SLE [121].

In conclusion, the diverse roles of KIR genes in SLE underscore their complex involvement in immune dysregulation and disease pathogenesis. Understanding these genetic variations and their interactions with HLA molecules provides valuable insights into the mechanisms underlying SLE susceptibility.

### 4.19. Systemic Sclerosis

Systemic Sclerosis (SSc) is a chronic autoimmune disease of the connective tissue characterized by abnormal immune system activation, vascular abnormalities, inflammation, and excessive extracellular matrix production, leading to skin and organ fibrosis. Previous studies have suggested associations between specific KIR genes and SSc susceptibility. For instance, KIR2DS2+/KIR2DL2- and KIR2DS3 have been implicated in SSc, indicating the potential roles of these genes in the pathogenesis [118,122,123]. Subsequent studies have not confirmed these findings. Conversely, Mahmoudi et al. highlighted the importance of KIR3DL1 with HLA ligands for diagnosing SSc [124].

In a study involving a southern Mexican mestizo population, KIR2DL2 was identified as a risk gene for SSc. The presence of KIR2DS4del further increases the risk, contrasting with KIR2DS4full, which shows a reduced risk association. Additionally, KIR/HLA compound genotypes, such as KIR2DL2+/HLA-C1+ and KIR2DL2+/HLA-C2, are implicated in SSc susceptibility, underscoring the complex genetic interplay in autoimmune pathogenesis [125]. However, a meta-analysis did not find significant variations in KIR polymorphisms between SSc cases and controls [126].

In conclusion, the role of KIR genes in SSc remains complex and somewhat contradictory across studies. Thus, further research in more extensive, diverse cohorts is essential to validate these associations and elucidate their potential clinical implications for SSc management and treatment strategies.

### 4.20. Type 1 Diabetes Mellitus

T1DM is an autoimmune disease where the immune system attacks and destroys the insulin-producing β cells in the pancreas, leading to hyperglycemia. Genetic predisposition to T1DM involves several factors, prominently including variations in the HLA genes, which are crucial for immune recognition. HLA class II genes, particularly HLA-DR3 and HLA-DR4 alleles, are well-established genetic risk factors for T1DM [127,128].

Studies on KIR genes have revealed significant associations with T1DM susceptibility across diverse populations. Initial findings suggested that dysregulation characterized by increased activation of KIR genes, without adequate inhibition, contributes to T1DM onset [129].

Research in East Indian populations highlighted specific KIR genes associated with T1DM, particularly KIR2DL2, consistent with findings from Latvian and Basque populations [130]. Conversely, studies in Korean and Han Chinese populations showed associations with KIR genes like KIR2DL5, KIR2DS2, KIR2DL1, KIR3DL1, and KIR2DS4, while other Asian studies did not find associations [131].

Subsequent studies emphasized the complex interactions between maternal KIR genes and HLA genes in T1DM susceptibility among the Chinese Han population. Maternal activating KIR genes were associated with increased T1DM risk in children, particularly those carrying the HLA-C2 gene, highlighting the prenatal influence on disease predisposition [132].

A comprehensive meta-analysis revealed no association between several KIR genes and T1DM susceptibility but suggested a protective role for KIR2DL1. It also found that KIR2DS1 was associated with a reduced risk of T1DM in Asians but not Caucasians [133]. A meta-analysis by Soltani S et al. confirmed the protective association of KIR2DL1 and identified KIR2DL5 as another protective gene, while KIR2DL2 was associated with increased risk [134].

Recent investigations also focused on the interplay of HLA ligands and KIR genes, revealing associations between specific KIR-HLA combinations and T1DM risk. Notably, HLA-C1C1 homozygosity was identified as a risk factor, whereas specific KIR genes like KIR2DS2, KIR2DL1, KIR2DL2, and KIR2DL3 showed protective effects in combination with HLA-C1C2 heterozygosity [135].

In conclusion, T1DM pathogenesis involves a complex interplay of genetic factors across different populations, including variations in HLA class I genes and KIR genes. Dysregulation of KIR gene expression, particularly in HLA interactions, influences immune responses implicated in β-cell destruction. Further studies are essential to elucidate the precise mechanisms underlying these genetic associations and their potential implications for personalized medicine in managing T1DM.

### 4.21. Vogt–Koyanagi–Harada Syndrome

The Vogt–Koyanagi–Harada syndrome (VKH) is a rare autoimmune disease characterized by inflammation of the eyes (uveitis), skin disorders, hair alterations, and involvement of other organs such as the nervous system and respiratory tract.

Research into the genetic factors associated with VKH has focused on KIR genes and HLA ligands. The first study showed an increased frequency of activating KIR genes KIR3DS1, KIR2DS1, KIR2DS2, and KIR2DS3, along with their presumed HLA ligands in patients with VKH compared to controls [136]. Subsequently, the same research team revealed that the presence of activating KIR receptors, particularly KIR3DS1, KIR2DS1, and KIR2DS5, along with the absence of the KIR3DL1, increases the risk of developing VKH in Japanese patients [137]. In 2011, Sheereen et al. suggested that KIR2DS3 and class I HLA molecules may play a role in VKH pathogenesis. Additionally, a predominance of KIR2DL2/KIR2DL3/HLA-C1 in the control group implied a potential protective role of KIR/ligand interaction against VKH development or severity [138].

A subsequent study revealed that VKH is associated with KIR2DL2, KIR2DS2, KIR2DS3, and KIR2DL5B. Their study contrasted earlier observations by indicating increased frequencies of KIR B haplotypes and specific activating KIR genes in VKH patients [139].

The study of KIR genes in VKH highlights their potential role in disease susceptibility and pathogenesis.

**Table 1:** KIR genes and autoimmune disease.

	Predisposing KIRs	Protective KIRs
Ankylosing spondylitis	KIR3DS1 [26,27] KIR2DS1 [27] KIR2DS4 [26] KIR2DS5 [26,27] KIR2DL5 [26,27] KIR3DL1 [26]	KIR2DL2 [27] KIR2DS2 [27]
Atopic dermatitis	KIR2DS1 [31,32] KIR2DL5 [31,32] KIR2DS5 [31,32] KIR2DL4 [32] KIR2DS4 [32]	KIR2DS1 [30]
Autoimmune hepatitis	KIR2DS1 [37] KIR2DS4 [38] KIR3DL1 with HLA-B Bw4-80Ile [39]	KIR2DL3 [37] KIR3DL1 with HLA-B Bw4-80Thr [37] KIR2DL1 with HLA-C2 [39] KIR2DL2 [38] KIR3DL1 [39]
Behçet’s disease	KIR3DS1 [45]	KIR3DL1 [46]
Celiac disease	KIR2DL5B [50,52] KIR3DL1 [51] KIR2DL2 with HLA-C1 [52] KIR2DS2 [52] KIR2DS3 [52] KIR2DL3 with HLA-C1 [53] KIR2DS5 [54] KIR3DS1 [54]	\
Graves’ Disease	KIR2DS2-, KIR2DL2-, KIR2DL3+, KIR2DL1+, KIR3DL1+, KIR3DS1-, KIR2DL5-, KIR2DS3-, KIR2DS5-, KIR2DS1-, KIR2DS4- [57]	KIR2DL1 with HLA-C2 [60]
Hashimoto’s thyroiditis	KIR2DS2 with HLA-C1 [63]	KIR2DS2-/KIR2DL2+/KIR2DL3+/HLA-C1 [63]
Immune thrombocytopenia	KIR2DS2 [65,66,67] KIR2DS3 [67] KIR2DL2 [65,66]	KIR2DS5 [67] KIR2DL3 [64] KIR3DL2 [64] KIR3DL1 [64]
Inflammatory bowel disease	KIR2DS2 [68] KIR2DL2 [68] KIR3DL1 with HLA-Bw4 [69] KIR2DL3 with HLA-C1 [70] KIR2DS3 [69] KIR2DL5 [71,73] KIR2DS1 [71,73]	KIR2DS3 [71] KIR2DL3 with HLA-Cw1 [68] KIR2DL2 [70] KIR2DS2 [70]
Juvenile Idiopathic Arthritis	KIR3DL1/KIR2DL2 [75]	KIR2DS4 [74]
Myasthenia gravis	\	\
Multiple Sclerosis	KIR3DS1 [80] KIR2DL5 [80] KIR2DL1 [80] KIR2DS5 [80] KIR2DL2/KIR2DS2 [84]	KIR2DS1 [86] KIR2DL3 [84] KIR3DL1 with HLA-Bw4 [85] KIR2DL2/KIR2DL3 with HLA-Bw4 [87]
Non-celiac wheat sensitivity	/	KIR2DL5 [89] KIR2DS4 [89] KIR2DS5 [87]
Psoriatic Arthritis	KIR2DL1 [9] KIR2DL2 [4,5,6,7,8,9,10,11,12,13,14,15,16,17,18,19,20,21,22,23,24,25,26,27,28,29,30,31,32,33,34,35,36,37,38,39,40,41,42,43,44,45,46,47,48,49,50,51,52,53,54,55,56,57,58,59,60,61,62,63,64,65,66,67,68,69,70,71,72,73,74,75,76,77,78,79,80,81,82,83,84,85,86,87,88,89,90] KIR2DS1 [90,91,92,93,94,95] KIR2DS2 [90,91,92,93,94,95] KIR2DS3 [90,91,92,93,94,95]	\
Psoriasis Vulgaris	KIR2DS1 [102] KIR2DS5 [102] KIR3DS1 [102]	KIR2DS4 [102] KIR3DL1 [102] KIR2DL1 [102]
Rheumatoid arthritis	KIR2DS2 [103,104] KIR2DL2 [104,109] KIR2DS4 [104,109] KIR2DL1 [105] KIR2DS1 [105]	KIR2DL3 [104,105,106,109] KIR2DL5A [104,109] KIR2DL5 [106] KIR2DS5 [106] KIR3DL3 [106] KIR2DL2/KIR2DS2 (treatment) [107]
Sjögren’s syndrome	KIR2DS2+/KIR2DL2- [111]	\
Systemic Lupus Erythematosus	KIR2DS1 [112,113,114,115,116] KIR2DS2 [112,113,118] with HLA-C1 [120] KIR2DL3 [119] KIR3DL1 [119] KIR2DL2 [115,116] KIR3DS1 [119] KIR2DL5B [120]	KIR2DL3 with HLA-C1 [119,120] KIR2DL5 [112] with HLA-Bw4 [119] KIR2DL5B with HLA-C1 [120] KIR3DL1 [119] KIR2DL2 with HLA-C1 [120] KIR2DS2 with HLA-C1 [120]
Systemic sclerosis	KIR2DS2+/KIR2DL2- [118,122,123] KIR2DS3 [118,122,123] KIR3DL1 [124] KIR2DS2 [122,123] KIR2DL2 with HLA-C1/C2 [125] KIR2DS4del [125]	KIR2DS4full [125]
Type 1 Diabetes Mellitus	KIR2DL2 [129,130,134] KIR3DL1 [134] KIR2DS4 [134] KIR2DL5 [131] KIR2DS2 [131] KIR2DL1 [131] KIR3DL1 [131] KIR2DS4 [131]	KIR2DL1 [133,134,135] KIR2DL5 [134] KIR3DL1 with HLA-C1C2 [135] KIR2DL3 with HLA-C1C2 [135] KIR2DS2 with HLA-C1C2 [135] KIR2DS1 with HLA-C1C2 [133]
Vogt–Koyanagi–Harada syndrome	KIR3DS1 [136] without KIR3DL1 [136] KIR2DS1 [136] without KIR3DL1 [136] KIR2DS2 [136,139] KIR2DS3 [136,138,139] KIR2DS5 without KIR3DL1 [137] KIR2DL2 [139] KIR2DL5B [139]	KIR3DL1 [137] KIR2DL2/KIR2DL3 with HLA-C1 [138]

## 5. KIR Genes and Cancer

The intricate balance between inhibitory and activating signals mediated by KIRs determines NK-cell tolerance versus activation. Inhibitory KIRs typically interact with self-HLA class I molecules to suppress NK-cell cytotoxicity, maintaining self-tolerance. However, many cancer cells evade cytotoxic T-lymphocyte responses by downregulating HLA class I expression. This strategy can paradoxically increase their vulnerability to NK-cell-mediated lysis due to reduced inhibitory KIR signaling. The outcome of KIR-HLA interactions in the tumor microenvironment is highly context-dependent and varies across cancer types. For instance, tumors such as colorectal and lung cancers often retain HLA class I expression, allowing engagement of inhibitory KIRs and facilitating immune evasion [140]. In contrast, malignancies with diminished HLA class I may provoke NK-cell activation, especially when activating KIRs and their ligands are present. Recent studies have also linked specific KIR genotypes and haplotypes to differential cancer susceptibility. For example, the presence of KIR2DL2 and KIR2DS2 has been associated with an increased risk of lung cancer, while KIR2DS5 has emerged as a risk factor for thyroid cancer [141]. Such findings suggest that certain KIR genes’ presence and expression levels contribute to the etiology of solid and hematologic malignancies. This growing body of evidence highlights the relevance of KIR diversity in immune surveillance and cancer development and progression, with significant implications for patient-specific risk assessment and therapeutic targeting [142,143]. In this section, we describe the evidence on the role of KIR polymorphisms in different cancers (Table 2).

### 5.1. Biliary Tract Cancer

Biliary tract cancers (BTCs) are rare and highly lethal, involving the hepatobiliary system. The two main subtypes of BTC are gallbladder cancer (GBC) and cholangiocarcinoma (CCA). These tumors, rich in NK cells, develop inside and near the liver [144]. Only a Swedish case-control study investigated the association between KIR genes and BTC. The authors found a lower prevalence of KIR2DL3 in patients with BTC compared to healthy controls [145]. Additionally, the KIR2DL2–HLA-C1 interaction was more prevalent in patients with BTC than healthy controls. Finally, the activating gene KIR3DS1 was found to be associated with GBC. The study highlighted that NK cells play a role in surveilling the biliary tree inside and outside the liver, potentially involving the KIR-HLA system. In summary, the results indicate that patients with BTC exhibit a genetically distinct architecture in their KIR-HLA locus compared to healthy controls, with potential implications for immune surveillance of tumors [145].

### 5.2. Bladder Cancer

Bladder cancer (BC) is one of the most common malignancies of the urinary tract, primarily affecting the urothelium [146]. It represents a significant public health concern due to its high incidence, especially in men; recurrence rates; and the need for lifelong surveillance. In 2018, an Iranian research group first explored the association between KIR genes and urothelial BC [147]. They found that the KIR2DL1 and KIR2DS4 genes were more common in BC patients than controls, while the KIR2DL2 and KIR2DS2 genes were less common. KIR2DL2 inhibits NK-cell activity, while KIR2DS2 is linked to inflammatory conditions associated with increased INF-γ production. The absence of KIR2DL2 leads to a lack of inhibitory signals, resulting in constant NK-cell activation and contributing to inflammation, which is essential for the development and progression of BC. Recently, Guillamón et al. compared KIR/HLA-ligand genotypes between 132 BC, 201 other solid cancers, 164 plasma cell disorders, and 615 healthy controls [148]. The authors showed that the absence of KIR2DL1 or the presence of HLA-C1 protects against BC, while KIR2DL5 is a predisposing factor. These findings highlight the importance of genetics in understanding immune surveillance of tumors and could pave the way for new personalized therapeutic strategies.

### 5.3. Breast Cancer

Breast cancer is the most frequently diagnosed malignant tumor in women worldwide [149]. It originates from the breast cells, typically in the ducts or the lobules. While most breast cancer cases are diagnosed in women, men can also develop the disease, albeit at a much lower rate. Several studies have shown that there is an association between the presence/absence of KIR genes and the risk of developing breast cancer. The first study showed that KIR-Bx genotypes increased significantly in BC than controls, and the increase was more pronounced in advanced cancer [150]. No difference was observed with inhibitory KIR and HLA–ligand combinations. The activating KIR and HLA–ligand combinations, KIR2DS1 /HLA-C2 and KIR3DS1 /HLA-Bw4, were significantly increased in advanced BC. Then, a study conducted by Alomar et al. demonstrated a protective effect of the KIR2DS2, KIR2DS3, and KIR2DL5A genes against BC [151]. Specifically, the synergistic action of the three genes was observed when they occurred together, and the absence of the three genes increased BC occurrence by 6.5-fold. The HLA-C1/C2 ligand distribution between patients and controls showed an increased risk of BC occurrence for the heterozygote C1/C2 and a protective effect of the homozygous C2/C2. Combinatory analyses of KIR genes and their HLA-C ligands showed protective effects of KIR2DL2 and KIR2DL3 without their HLA-C1 ligand. These results suggested that KIR gene content combined with their ligand could influence the risk of BC development. A subsequent study showed that the rate of activating KIR2DS1 was much higher in patients with BC than in healthy controls. In contrast, the allelic types of activating KIR2DS4 (KIR2DS4 003/4/6/7) were lower in patients with BC than in healthy controls [152]. Additionally, there was a negative correlation between the KIR2DL1 gene and BC development. This study suggests that the activating KIR2DS1 may trigger BC development, while the KIR2DL1 gene and KIR2DS4 003/4/6/7 alleles are possibly protectors. Jobim and colleagues observed that the presence of inhibitory KIR2DL2 receptors was significantly higher in BC patients than in healthy controls [153]. No significant differences were found for HLA-C2 and HLA-Bw4. In a study on a cohort of Iranian women, the authors observed a lower frequency of KIR2DL1 and KIR2DS4del in BC than in the control group [154]. Further analysis revealed a higher frequency of KIR2DL2, KIR2DS1, KIR2DS2, KIR3DS1, KIR2DL5, and KIR2DL1 in BC compared with controls. Furthermore, the authors noted the predisposing role of the Bx genotype, KIR2DS1, KIR2DS2, KIR2DS5, KIR2DL2, and KIR2DL5 for lymphatic invasion, with a higher rate of lymph node metastasis. More recently, Canossi et al. highlighted a reduction of KIR2DS4 in BC patients and an increased combined presence of KIR2DL1 and KIR2DS1 genes in advanced BC patients compared to earlier stages [155]. The concurrent lack of KIR2DL2 and KIR2DS4 genes in the presence of HLA-C2 alleles was significantly associated with increased susceptibility to BC or lymph node involvement.

The heterogeneity among studies is likely due to multiple factors, including ethnic and population disparities, different BC histological phenotypes, and small sample sizes. Thus, further studies are mandatory to evaluate the role of KIR genes in BC.

### 5.4. Cervical Neoplasia

Cervical neoplasia (CN) is characterized by the abnormal and uncontrolled growth of cells in the cervix. This condition is often associated with persistent infections by certain human papillomavirus (HPV), one of CN’s leading causes [156].

The first case-control study investigating the association between KIR genes and CN found that KIR2DL1, KIR2DL2, KIR2DL3, KIR2DL4, KIR3DL1, KIR3DL2, KIR2DL3, and KIR2DS4 are associated with an increased CN risk, while the KIR2DL5 is protective [157].

In a study conducted on a cohort of Indian women affected by HPV, some with untreated CN, a high frequency of activating KIR genes, such as KIR2DS1, KIR2DS2, KIR2DS3, KIR2DS4, KIR2DS5, and KIR3DS1, was found in all patients. Specifically, KIR2DS5 was more prevalent in women with CN (83.3%), significantly higher than in healthy controls and women with HPV infection without neoplasia. Among inhibitory KIR genes, the frequency of KIR2DL1 was higher in healthy controls than in CN, and the frequency of KIR2DL5 was higher in healthy controls than in women with HPV infection. The frequency of KIR2DL3 was higher in women with HPV infection compared to those with CN [158].

A study conducted in 2018 found no association between KIR genes and the development of CN but demonstrated that HLA-Bw4 alleles were associated with an increased risk of HPV16-related CN. This association was limited to KIR3DL1 carriers. Conversely, the protective association against HPV16-related cervical neoplasia of the HLA-C1/C1 allele was limited to individuals carrying KIR2DL2 or KIR2DS2. No association was observed between HPV16-related CN and individuals with KIR2DS1 [159]. Consistent with these findings, an Australian cohort study demonstrated weak associations between KIR2DL2 and KIR2DS2 and cervical intraepithelial neoplasia [160]. Conversely, a study conducted on individuals from the eastern United States and Costa Rica found that the frequency of KIR3DS1 increased in patients with CN [161]. A study conducted on a population of Chinese women with cervical intraepithelial neoplasia revealed that, among various inhibitory KIR genes, the frequency of KIR3DL1 was higher in healthy controls compared to CN, suggesting its protective role [162].

Based on literature evidence, not only persistent cervical HPV infection but also KIR genes may predispose women to CN.

### 5.5. Colorectal Cancer and Metastatic Colorectal Cancer

Colorectal cancer (CRC) is one of the most common malignancies worldwide, affecting the colon and rectum. It ranks as the third most frequently diagnosed cancer and the second leading cause of cancer-related deaths. CRC develops slowly over many years, often starting from small non-cancerous polyps on the inner wall of the colon or rectum. Over time, some of these polyps can become cancerous and develop into tumors [163].

Several studies have examined how different KIR genes might influence CRC risk. In 2014, one of the first studies showed that KIR2DL5, KIR2DS5, KIR2DS1, and KIR3DS1 expression and KIR2DS4 and KIR3DL1 absence are associated with an increased risk of developing CRC [164]. In the same year, Kim et al. showed that the frequency of KIR3DS5 is higher in Korean patients with CRC, while the frequency of KIR3DL1, KIR3DS2, and KIR2DS4 is lower, suggesting its protective role [165].

A study by Ghanadi and colleagues found that the KIR2DS5 gene is present in all patients with CRC but not in healthy controls. No protective KIR gene was found, suggesting that inflammation may play a significant role in the development of this cancer [166].

A study conducted on a Brazilian Caucasian population with CRC did not confirm the role of KIR2DS5 in CRC predisposition [167]. Later, it was shown that the KIR2DS1, KIR2DS5, KIR3DS1, KIR2DS4, and KIR2DL5 genes predispose to CRC development. However, these KIR genes also lead to resistance to metastasis. This controversial finding might be due to NK cells enhancing the expression of inhibitory receptors that counteract CRC metastasis [168]. Recently, it was shown that the frequency of KIR2DS3 was significantly increased in patients with CRC compared to healthy controls [169].

In conclusion, several studies have shown that some KIR genes (e.g., KIR2DS5, KIR2DS1, KIR3DS1) are associated with an increased risk of developing CRC, while others (e.g., KIR2DS4, KIR3DL1) might have a protective role. The contradictions in the results indicate the need for further research to understand better these relationships and the role of NK cells in the disease and resistance to metastasis.

### 5.6. Dermal Neurofibroma

Dermal neurofibroma (DNF) is a tumor derived from Schwan’s peripheral nervous system cells, typically detected in neurofibromatosis type 1 (NF1). It is commonly a benign tumor manifesting with café-au-lait spots and cutaneous neurofibromas. NF1 is a genetic disorder involving the nervous system, with DNFs being the main feature [170].

Only one study investigated the relationship between KIR genes and the risk of DNFs, showing that one-third of patients with spontaneous DNFs have a mutation in KIR2DL5N173D, resulting in reduced gene expression [171]. Additionally, reduced activity of the KIR2DL5 gene was associated with increased cell proliferation. Silencing KIR2DL5 gene RNA was linked to the overactivation of certain cellular signaling pathways involved in tumor formation. These findings suggest that the KIR2DL5 gene plays a key role in regulating the growth of Schwann cells involved in developing spontaneous DNFs through excessive activation of a signaling pathway called the RAS pathway. Further research is needed to fully understand the genetic and molecular mechanisms underlying DNFs and develop potential therapeutic strategies.

### 5.7. Hepatocellular Carcinoma

Hepatocellular carcinoma (HCC) is the fifth most common cancer globally. Risk factors such as male gender, advanced age, cirrhosis, alcohol consumption, and infection with hepatotropic viruses like hepatitis C (HCV) and hepatitis B (HBV) are often linked to HCC development [172].

Pan and colleagues identified an association of KIR2DS4 activating a mutant variant, called KIR2DS4/1D, with HBV-associated HCC. Specifically, KIR2DS4/1D results in a truncated and functionally inactive form of the KIR2DS4 protein [173].

A study on an Italian cohort of patients with HCV infection revealed that patients lacking activating KIR genes have a higher risk of developing HCC [174]. Specifically, in contrast to the first study, some activating KIRs, such as KIR2DS4/1D, KIR2DS1, KIR2DS2, and KIR3DS1, were less frequent in HCC patients. A study conducted on HCC patients of Chinese ethnicity found that the frequency of KIR2DL3 is significantly lower in patients than in healthy controls [175].

A study on an Egyptian cohort found that the KIR AA haplotype, with more inhibitory KIR genes and fewer activating genes, is less frequent in HCC patients compared to those with HCV and healthy controls [176]. However, KIR2DS1 and KIR3DS1 are higher in HCC patients, and the presence of KIR2DL5 and KIR2DS5 is more increased in HCC patients than those with chronic HCV and healthy controls.

There are clear differences in KIR gene frequencies among various ethnicities and in relation to specific viral infections, suggesting a complex role of these genes in predisposing and developing HCC. Further research is needed to understand how KIR genes influence susceptibility to HCC and to develop targeted preventive and therapeutic strategies.

### 5.8. Kidney Cancer

Kidney cancer (KC) affects nearly 300,000 individuals worldwide each year and is responsible for more than 100,000 deaths annually. The most common type of KC is renal cell carcinoma (RCC), which accounts for most cases [177].

Only one study explored the possible association between KIR genes and the risk of developing solid tumors [178]. Specifically, in patients with KC, a significantly reduced frequency of KIR2DL2 and KIR2DL3 and their ligand HLA-C1 was detected. Conversely, an increased frequency of KIR3DL1 and its ligand HLA-Bw4 compared to controls was found. Furthermore, it was demonstrated that KC patients undergoing transplantation with more activating KIR genes are significantly protected from cytomegalovirus infection. These findings show an increased co-expression of inhibitory KIR and their ligands, resulting in decreased function of NK cells that may play a protective role in KC development.

### 5.9. Leukemia

The role of KIR genes has been explored in different types of leukemia, including acute lymphoblastic leukemia (ALL), chronic lymphocytic leukemia (CLL), acute myeloid leukemia (AML), and chronic myeloid leukemia (CML).

ALL, the most common form of leukemia in children, results in an abnormal increase in immature lymphocytes. CLL is a slow-growing cancer that mainly affects older adults. Myeloid leukemia causes the rapid growth of myeloid cells; its acute form (AML) occurs either in children or in adults, and its chronic form (CML) affects primarily adults [138].

The first case-control study describing an association between polymorphisms of KIR genes and leukemia showed that leukemia patients have an increased frequency of the KIR AB phenotype [179]. The AB phenotype is characterized by two genes encoding for KIR2DL2 and KIR2DL3 and one gene encoding for KIR2DL1. The frequency of the inhibitory KIR2DL2 was significantly higher in leukemia patients than in controls. The results of this study show that NK cells in leukemia patients are inhibited due to the presence of many inhibitory KIRs.

Middleton and colleagues analyzed the frequency of KIR in cohorts of Turkish patients with ALL, AML, and CML [180]. Specifically, the authors observed that the co-expression of KIR2DL2 and/or KIR2DS2 with the HLA-C1 ligand protects against the risk of leukemia, while with the HLA-Bw4 ligand is associated with an increased risk of leukemia. Then, Zhang and colleagues observed that the frequency of KIR2DS4 was significantly higher in patients with CML compared to healthy controls, while KIR2DS3 was lower in patients with ALL compared to healthy controls [181]. This finding indicates that KIR2DS4 might be a susceptibility gene for leukemia, while KIR2DS3 may be protective.

A case-control study on Canadian children with pre-B ALL (B-ALL) showed that an increased frequency of activating KIR genes, especially the KIR2DS2 gene, is associated with a reduced risk of developing B-ALL [182].

Babor and colleagues conducted a similar analysis in a cohort of European pediatric patients with B-ALL to assess the possible role of KIR2DL2, which is in strong linkage disequilibrium with KIR2DS2 [183]. However, unlike the previous study, the authors found no positive association between KIR2DL2 and the risk of B-ALL.

Some authors observed differences in KIR genes among different subtypes of leukemia. For example, it was shown that KIR2DS3 is more frequent in AML compared to ALL patients [184]. Additionally, the inhibitory KIR2DL2 and KIR2DL5 and the activating KIR2DS1 and KIR2DS2 are more frequent in healthy controls. In contrast, Al-Taminimi et al. found that ALL patients had an increased frequency of the two inhibitory KIR genes, 2DL1 and 3DL1, and the activating 2DS4 compared to healthy controls [185].

Smith and colleagues demonstrated that the frequency of the KIR AA genotype significantly increases in ALL pediatric patients compared to controls [186]. Specifically, stratifying the study population by ethnicity, a significant difference in the frequency of the KIR genotype was found between Hispanic and European patients. Homozygosity for HLA-Bw4 was strongly associated with an increased risk of ALL exclusively in European children. Therefore, the role of KIR genes and their HLA ligands in the etiology of childhood ALL may vary among ethnic groups.

Then, a recent study demonstrated that the KIR genotype frequency differed significantly between myelogenous leukemia patients and healthy controls [187]. Specifically, the KIR genotype characterized by the absence of the inhibitory KIR2DL2 and the activating KIR2DS2 and KIR2DS3 was found at a lower frequency in patients than controls. Similarly, Cianga et al. showed that AML patients have increased frequency of the inhibitory AA KIR haplotype and decreased 2DS3 [188].

A study on a population of 1767 German AML patients and 51,890 healthy controls found no significant differences in the presence or absence of individual KIR genes [189]. There was no significant difference in the frequency of KIR2DL1, KIR2DL2, KIR2DL3, KIR2DL5, KIR2DP1, KIR2DS1, KIR2DS2, KIR2DS3, KIR2DS4, KIR2DS5, KIR3DL1, and KIR3DS1 genes between patients and control subjects. For example, KIR2DS4 is present in 96.4% of patients with AML and 95.1% of the control subjects.

Some studies demonstrated that variants of KIR genes influence the susceptibility to or progression of leukemias. However, the data available in the current literature are still inconclusive and sometimes contradictory, indicating the need for further studies to clarify the precise role of KIR genes in the different forms of leukemia.

### 5.10. Lung Cancer

Lung cancer (LC) is characterized by the uncontrolled growth of abnormal cells in the lung tissue. LC is the most diagnosed cancer worldwide, accounting for approximately 12.4% of all cancers, and is the leading cause of cancer-related deaths [190]. It is classified into two main histological subtypes, small cell lung cancer (SCLC) and non-small cell lung cancer (NSCLC) [191], which is most common in the general population [192]. Similarly to other neoplastic diseases, NSCLC is a multifactorial disease due to both environmental (mainly cigarette smoking) and genetic factors, including KIR genes [192]. Some authors have explored the association between KIR and their HLA ligands in the risk of developing LC, specifically NSCLC. Li Y et al. reported no differences in the KIR genotype and haplotype frequencies between the NSCLC patients and control groups [193], while Ty et al. observed that the expression of KIR2DS4del was associated with a significantly decreased survival time in metastatic NSCLC in a Chinese population [194]. In the Polish NSCLC patients, Wiśniewski and colleagues reported an association between KIR2DL2 and KIR2DS2 with more prolonged survival and a better response to chemotherapy [192]. This result contrasts with a recent study showing that KIR2DL2 and KIR2DS2 increase the risk of developing LC in combination with HLA-C1 ligands [195]. Furthermore, KIR3DL1 and KIR3DS1 were less frequent in LC patients. This discrepancy among studies may be because of chemotherapy agents or the expression of ligands that activate or inhibit NK cells. Variations in KIR genes and their HLA ligands show conflicting associations with the risk and progression of LC.

### 5.11. Melanoma

Melanoma is a skin cancer caused by a malignancy of melanocytes. The incidence of melanoma is rapidly increasing worldwide, representing the fifth and sixth most common type of cancer in men and women, respectively [196]. The relationship between exposure to ultraviolet (UV) light and melanoma development is complex, and intermittent sun exposure dramatically increases the risk of melanoma. It is known that NK cells play a role in immunosurveillance in melanoma progression through the interaction between KIR receptors and their HLA ligands [197].

The first association study observed an increased frequency of KIR2DL2 and KIR2DL3 in combination with the HLA-C ligand and an increased frequency of KIR2DS1 in the absence of the HLA-C ligand in patients with melanoma [198]. Additionally, the authors observed an increased incidence of KIR3DL1 and the HLA-Bw4Ile80 ligand in patients with metastatic disease compared to patients with primary tumors. In contrast, Campillo JA et al. found a low frequency of KIR2DL3 in nodular melanoma patients and ulcerated melanoma patients [197]. Moreover, the KIR2DL3/HLA-C1 combination decreased in patients with melanoma and sentinel lymph node melanoma metastasis. Kandilarova SM et al. observed that KIR2DS5 showed a reduced frequency in patients with rapid progression compared to those with slow progression [199]. Additionally, the KIR BB genotype was prevalent in patients with metastasis, while the KIR AA genotype was more frequent in rapidly progressive cases, but without statistical relevance. A significantly increased frequency of KIR2DL2 in the presence of the HLA-C1 ligand was found in patients with advanced melanoma compared to individuals with early-stage melanoma. Despite these findings, it is necessary to continue further research to understand how KIR genes and their ligands are involved in the various stages of melanoma.

### 5.12. Multiple Myeloma

Multiple myeloma (MM) is a type of blood cancer characterized by the abnormal accumulation of plasma cells in the bone marrow [200]. Studies have shown that NK cells have antitumor activity against MM [201]. In 2007, Jurisic and colleagues investigated the association between KIR genes and MM for the first time, finding that advanced stages of MM were associated with reduced NK-cell activity [202]. Hoteit and colleagues confirmed an association between KIR genes and Lebanese MM patients, identifying a higher prevalence of KIR2DS4 and KIR2DS5 genes [203]. However, the overall distribution of KIR genotypes did not differ between MM patients and healthy controls. A recent association study conducted in a Thai MM population showed that the frequencies of KIR3DL1 and KIR3DS4 were significantly lower in MM patients compared to healthy controls, suggesting a protective role. On the other hand, the frequencies of KIR3DL1, KIR2DS4, and KIR2DL1 were significantly higher in MM patients than in controls [204]. Beelen and colleagues also observed increased KIR3DL1 gene in MM patients [205]. These results suggest that specific KIR genes may influence the susceptibility and progression of MM, providing potential targets for future therapies.

### 5.13. Mycosis Fungoides

Mycosis fungoides (MF) is the most common cutaneous T-cell lymphoma. MF is characterized by the clonal expansion of CD4+ T-helper lymphocytes in the skin. However, infiltrates of cutaneous tumors contain a considerable number of CD8+ T cells and NK cells. The regulation of NK-mediated cytolysis may be altered by combining KIR receptors and HLA ligands [206]. The only study examining an association between KIR genes and MF investigated a cohort of 46 Caucasian patients. Specifically, the authors demonstrated that the frequency of HLA-A ligands for KIR3DL2 (HLA-A3 and -A11) was lower in MF patients than in healthy controls. Conversely, the frequency of HLA-A ligands for KIR3DL1 was higher in MF patients than in healthy controls.

So far, the association of HLA alleles and KIR with the prognosis of the disease has not been thoroughly analyzed. The authors attempted to assess whether the absence or presence of specific HLA ligands for KIR could be involved in the prognosis of MF. They also observed that the presence of the HLA-DQB1*05 allele distinguishes patients with MF and the most unfavorable prognosis [207].

### 5.14. Neuroblastoma

Neuroblastoma is a type of tumor developing in neuroblasts, immature nerve cells in the sympathetic nervous system. This tumor can manifest in the spine, chest, abdomen, or other areas of the sympathetic nervous system. Some neuroblastomas may regress spontaneously or respond well to treatment, while others can be more aggressive and difficult to treat [208]. Several studies have investigated the role of KIR genes and their HLA ligands, especially in response to antibody immunotherapy [209,210]. Keating et al. observed an increase in the KIR2DL2 and KIR2DS2 genes in patients with neuroblastoma compared to healthy controls [211]. Additionally, patients with the KIR2DS3 genotype showed increased cellular cytotoxicity against neuroblastoma [212]. A recent study observed that the inhibitory genes KIR2DL1 and KIR3DL1 had high frequencies in patients with neuroblastoma. There was a significant increase in the expression of the inhibitory gene KIR2DL3 and a significant decrease in the expression of the activating gene KIR2DS3 in the patient group compared to healthy controls. Additionally, patients with early-stage neuroblastoma showed higher expression of KIR2DS3 and lower expression of KIR2DL3 compared to patients with metastatic disease. Although the patient sample was small, these results suggest that KIR2DL3 and KIR2DS3 may play a role in neuroblastoma development [208]. Studies on KIR genes in neuroblastoma suggest an essential interaction between KIR receptors and their HLA ligands, potentially influencing the disease’s development and progression. However, further research is needed to fully understand the specific role of KIR genes and their clinical implications in neuroblastoma treatment.

### 5.15. Non-Hodgkin Lymphoma

Non-Hodgkin lymphoma (NHL) is a neoplasm of the lymphoid tissues originating from B-cell precursors, mature B cells, T-cell precursors, and mature T cells [213]. NHL comprises various subtypes, including diffuse large B-cell lymphoma (DLBCL), the most common type of NHL. In 2015, an association was demonstrated between polymorphisms of KIR genes, their ligands, and susceptibility to NHL. Specifically, the authors described that the ligands of KIR2DS1, HLA-Bw4 (Thr80), and HLA-Bw4 (Thr80)+/Bw4 (Iso80)- were significantly more frequent in NHL patients. The genotypes KIR2DL5A, KIR2DS1, and KIR3DS1 in patients with advanced-stage NHL contributed to an unfavorable prognosis. Patients with advanced-stage NHL had more frequent KIR2DL5 and KIR2DL5B than those with early-stage NHL [214].

In 2023, Daniela Maira C. and colleagues demonstrated a significant association between KIR genes and their HLA ligands with the clinical course of the disease. Specifically, an association of the HLA-Bw4 and HLA-Bw480I ligands with more advanced stages of the disease was found. Additionally, an association of the KIR2DL3 gene with a better response to treatment was identified, suggesting its protective role [215]. Therefore, the polymorphism of KIR genes and the association with HLA ligands can influence the disease’s prognosis and the response to treatment.

### 5.16. Non-Melanoma Skin Tumors

Non-melanoma skin cancers (NMSCs) include basal cell carcinoma (BCC), squamous cell carcinoma (SCC), and Merkel cell carcinoma (MCC). These tumors are heterogeneous in clinical presentation and biological evolution [216]. It has been shown that the function of NK cells is compromised in these types of tumors, suggesting their critical role in the development and progression of NMSC [217].

A study conducted on 160 Iranian patients found a significantly higher frequency of KIR2DL1 in patients with BCC than in healthy controls. In particular, the authors observed that combining KIR3DL1 with HLA-Bw4 (T80) was more frequent in patients with primary cutaneous tumors than in those with metastasis. This is because KIR3DL1 generates a weaker inhibitory signal. Overall, inhibitory KIRs reduce the anti-tumor response of NK cells. Furthermore, the authors found that KIR2DS4 was more frequent in patients with BCC. KIR2DS4 is an activating receptor that stimulates NK cells, causing the secretion of IFN-γ [218]. However, this study did not confirm the findings of Vineretsky KA et al. They showed that the activating gene KIR2DS3 with HLA-C1 was associated with a twofold increase in the risk of developing BCC and SCC. The authors also tested the hypothesis that KIR and HLA class I polymorphisms may play a role in the development of keratinocyte cancer and could exert selective pressure to alter p53 [219]. Understanding the interactions between KIR gene polymorphisms and HLA ligands and their influence on NK-cell function is crucial to outlining new diagnostic and therapeutic approaches for NMSC patients. Future research should aim to clarify these mechanisms further to develop personalized treatment strategies that can improve the prognosis and treatment response in these patients.

### 5.17. Ovarian Cancer

Ovarian cancer (OC) is a neoplasm that develops in the ovary’s epithelial cells. Depending on the type of epithelial cells involved, it can be classified as severe, mucinous, endometrioid, or clear-cell carcinoma [220].

A pioneering study on the association between KIR genes and the risk of developing OC revealed a significant association with the KIR2DS4 genotype, which was twice more frequent in patients with the endometrioid subtype compared to other ovarian cancers and healthy controls. However, this association was not observed in different cancer subtypes [221]. However, further research is needed to fully understand the complexity of this association and its impact on clinical practice.

### 5.18. Thyroid Cancer

Thyroid cancer (TC) is the most common endocrine tumor. It has a multifactorial origin involving the interaction between genetic and environmental factors, such as exposure to radiation and iodine deficiency in the diet. Ashouri and colleagues were the first to investigate the association between KIR genes and the risk of developing TC. The authors did not find significant differences in the frequencies of inhibitory KIR genes between patients and controls. Among activating receptors, KIR3DS1, KIR2DL5, KIR2DS1, and KIR2DS5 were significantly increased in patients with TC compared to healthy controls. Additionally, it was demonstrated that KIR2DS5 predisposes to an early onset of cancer and an increase in lymph node metastasis [222]. These receptors influence NK-cell activity, promoting the secretion of pro-inflammatory molecules and growth factors that facilitate tumor development and metastasis. Identifying these mechanisms could pave the way for targeted therapies and improve clinical management of thyroid cancer.

**Table 2:** KIR genes and cancer.

	Predisposing KIRs	Protective KIRs
Biliary tract Cancers	KIR2DL2 [145] KIR3DS1 [145]	KIR2DL3 [145]
Bladder Cancer	KIR2DL1 [147,148] KIR2DS4 [147] KIR2DL5 [148]	KIR2DL2 [147] KIR2DS2 [147]
Breast Cancer	KIR-Bx genotype [150,154] KIR2DS1 [150,154,155] KIR3DS1 [150,154] KIR2DS2 [159] KIR2DL2 [153,154] KIR2DL5 [154] KIR2DS5 [154]	KIR2DS2 [151] KIR2DS3 [151] KIR2DL5A [151] KIR2DL2 without HLA-C1 [156] KIR2DL3 without HLA-C1 [151] KIR2DL1 [152,154] KIR2DS4 (alleles 2DS4 003/4/6/7) [152,154,155]
Cervical neoplasia	KIR2DL1 [157] KIR2DL2 [157] KIR2DL3 [157,158] KIR2DL4 [157] KIR2DS2 [158] KIR2DS3 [158] KIR2DS4 [158] KIR2DS5 [158] KIR3DS1 [158,161]	KIR2DL5 [157] KIR3DL1 [167] KIR2DL2 [159,160] KIR2DS2 [159,160] KIR2DL1 [158]
Colorectal cancer and Metastatic Colorectal Cancer	KIR2DS1 [164,168] KIR2DS5 [164,166,167,168] KIR3DS1 [164,168] KIR3DS5 [165] KIR2DS3 [169] KIR2DL5 [164,168] KIR2DS4 [168]	KIR2DS4 [164,165] KIR3DL1 [164,165] KIR3DS2 [165]
Dermal neurofibroma	KIR2DL5 (N173D) [171]	
Hepatocellular carcinoma	KIR2DS4/1D [173] KIR2DS1 [176] KIR3DS1 [176] KIR2DL5 [176] KIR2DS5 [176]	KIR2DS4/1D [174] KIR2DS1 [174] KIR2DS2 [174] KIR3DS1 [174] KIR2DL3 [175] KIR AA Haplotype [176]
Kidney cancer	KIR3DL1 [178]	KIR2DL2 [178] KIR2DL3 [178]
Leukemia	KIR AB genotype [179] KIR2DL2 [179] KIR2DS4 [181] KIR3DL1 with HLA-Bw4 [180] KIR AA genotype [185] KIR2DS3 [181]	KIR2DL2 with HLA-C1 [180,181,186] KIR2DS2 with HLA-C1 [180] KIR2DS2 [182,186] KIR2DL2 [184,186] KIR2DL5 [184] KIR2DS1 [184] KIR2DS2 [179,186] KIR2DS3 [179,181,187]
Lung Cancer	KIR2DL2 with HLA-C1 [195] KIR2DS2 with HLA-C1 [195] KIR2DS4del [194]	KIR2DL2 [192] KIR2DS2 [192] KIR3DL1 [195] KIR3DS1 [195]
Melanoma	KIR3DL1 with HLA-Bw4Ile80 [198] KIR2DL2 with HLA-C1 [199] KIR BB genotype [199] KIR2DS5 [199]	KIR2DL2 with HLA-C [198] KIR2DL3 with HLA-C [198] KIR2DS1 without HLA-C [198] KIR2DL3 [197] KIR2DL3 with HLAC1 [197] KIR AA genotype [199]
Multiple Myeloma	KIR2DS4 [203,204] KIR2DS5 [203] KIR2DL1 [204] KIR3DL1 [205]	KIR3DL1 [204] KIR3DS4 [204]
Mycosis Fungoides	KIR3DL1 [207]	KIR3DL2 [207]
Neuroblastoma	KIR2DL2 [211] KIR2DS2 [211] KIR2DL1 [208] KIR3DL1 [208] KIR2DL3 [208]	KIR2DS3 [208,212]
Non-Hodgkin Lymphoma	KIR2DS1 [214] KIR2DL5 [214] KIR3DS1 [214] KIR2DL5A [214] KIR2DL5B [214]	KIR2DL3 [215]
Non-melanoma skin tumors	KIR2DL1 [218] KIR2DS4 [218] KIR2DS3 with HLA-C1 [219]	KIR3DL1 with HLA-Bw4 [218]
Ovarian cancer	KIR3DS4 [221]	/
Thyroid cancer	KIR3DS1 [222] KIR3DL5 [222] KIR2DS1 [222] KIR2DS5 [222]	/

## 6. KIR Genes and Infectious Diseases

NK cells play a role in the defense against infections by killing infected cells or producing cytokines and interacting with adaptive immune cells [223]. Specific KIR-HLA combinations can predict the outcome of or susceptibility to infections and facilitate the development of personalized therapeutic strategies [224]. KIRs can play a role in eliminating different microorganisms and protecting individuals by controlling a particular pathogen. In this section, we will explore the role of KIRs in infectious diseases and how these interactions may contribute to resistance or susceptibility to infections (Table 3).

### 6.1. Cytomegalovirus

Cytomegalovirus (CMV) is a DNA virus belonging to the Herpesviridae family. It infects newborns and adults, transmitted through infected bodily fluids or organ transplants. Moreover, CMV can also be transmitted to the fetus by infected pregnant women, either during pregnancy or childbirth. After primary infection, it remains latent but can reactivate in immunosuppressed conditions, causing severe symptoms such as pneumonia, hepatitis, retinitis, and encephalitis. The interaction between CMV and KIRs is crucial in determining the susceptibility and immune response in primary and reactivated CMV infections, particularly in immunocompromised individuals and transplant recipients.

Charoudeh et al. reported an expansion of NK cells expressing KIR2DL1, KIR2DL3, and KIR3DS1 in response to in vitro exposure to CMV [225]. In CMV seronegative donors, the observed changes were minimal in the subset of NK cells expressing the NKG2C antigen. The expansion of inhibitory KIR receptors was detected only in donors with specific class I HLA ligands, while the expansion of NK cells expressing KIR3DS1 did not require the presence of the putative HLA-Bw4 ligand. In contrast, Djaoud and colleagues found that during CMV infection in seropositive individuals, the inhibitory receptor KIR2DL3 is co-expressed with NKG2C on expanded NK cells, regardless of HLA-C expression [226]. Furthermore, these NK cells often also express KIR3DL1 or KIR3DS1, regardless of the presence of HLA-Bw4, suggesting a potential role of these receptors in the control of CMV infection.

The study of Béziat and colleagues demonstrated that CMV infection impacts the KIR variants of NK cells [227]. In particular, the authors observed an expansion of NK cells expressing activating KIRs (KIR2DS2, KIR2DS4, and KIR3DS1) in CMV seropositive donors, regardless of NKG2C. Moreover, Di Bona et al. found that carriers of the A homozygous haplotype or the HLA-Bw4T allele are more susceptible to developing symptomatic acute diseases after primary infection [228]. The influence of HLA-Bw4T on disease outcome could be mediated by the interaction of the allele with the inhibitory receptor KIR3DL1. The effect of HLA-Bw4T has been observed almost exclusively in individuals with the Bx haplotype, suggesting reduced protection associated with this genotype.

However, a subsequent study in a Sicilian population did not confirm this association, highlighting an association between the KIR2DS5 gene and the risk of symptomatic infection. In contrast, the KIR2DS2 gene is associated with a lower risk [229].

Crespo et al. evaluated the role of KIR genes in placental CMV infection [230]. In particular, the authors observed that the expression of KIR2DS1 in decidual NK cells enhances the ability to respond to placental CMV infections. In contrast, women lacking KIR2DS1 may exhibit a reduced capacity to control this infection, potentially leading to complications. These findings indicate a protective role of KIR2DS1 against placental CMV infection.

Further studies have evaluated the role of KIR genes in regulating CMV infection after transplantation. Indeed, it is well known that CMV is one of the most common infections after transplantation, posing a significant threat to the survival of recipients. Donors with activating KIR genes, such as KIR2DS2 and KIR2DS4, had a lower likelihood of CMV reactivation in stem cell transplants [231,232]. In a cohort of 447 recipients after liver transplantation, Wang et al. showed a negative correlation between the expression of activating KIR gene and the CMV infection [233]. Additionally, the authors showed that patients with KIR haplotype group B received protection against CMV infection after liver transplantation. However, these findings contrast with those of Moroso et al., who found that KIR genotypes did not predict the survival of liver transplantation recipients [234]. Thus, the literature on the influence of KIR on liver transplantation is contradicting and still needs further investigation [235]. Hadaya et al. observed that the absence of HLA ligands for inhibitory KIR receptors and many activating KIR receptors reduce the risk of CMV reactivation after kidney transplantation [236]. A study analyzed CMV viremia in solid-organ transplant patients with CMV serology donor positive/recipient negative [237]. The authors observed that KIR2DL3 and KIR2DS2 are strongly associated with the time to CMV viremia onset. Additionally, they evaluated the presence of the HLA-C1 ligand for these KIR genes. The study shows that the protective effect associated with the combination of KIR2DL3+/KIR2DS2+/HLA-C1/1 in transplant recipients was not observed if the transplanted organ came from a donor who did not have the HLA-C1/1 antigen. This suggests that the protective effect requires the presence of HLA-C1/1 in both the donor and the recipient.

In patients with the KIR A/A genotype (with KIR2DS4 as the only activating KIR), the CMV infection and reactivation rate was higher compared to transplant recipients with more than one activating KIR gene [238]. This protection was dose-dependent, and the more activating KIR genes an individual had, the lower the likelihood of CMV-related infectious episodes. A subsequent study highlighted that the telomeric complex of the B haplotype, containing genes such as KIR2DS1, KIR3DS1, KIR2DL5A, and KIR2DS5, may confer protection against CMV infection after kidney transplantation [239]. Conversely, Jones and colleagues indicated that an increase in these genes might be associated with a high viral load, while the A haplotype and HLA-C1 may be protective [240]. KIR2DS1 has been associated with protection against CMV reactivation both after hematopoietic stem cell transplantation (HSCT) [241] and kidney transplantation [242]. Gao et al. highlighted that KIR2DS1, KIR2DS3, and KIR3DS1 offer protection against the reactivation of CMV in patients with lymphoproliferative diseases. At the same time, these KIRs are associated with an increased risk of CMV reactivation in patients with myeloproliferative diseases [243]. Moreover, grafts lacking Tel B/x and KIR2DS5 in myeloid disease were associated with a lower risk of CMV reactivation.

Chen et al. indicated that KIR2DS2 in donors and HLA-C2 in recipients may increase the risk of reactivation [244], although other studies have suggested a protective role for KIR2DS2 alongside KIR2DS4 [232,245]. In contrast, another study found no associations with KIR2DS2 but found that KIR2DL3 and KIR2DL2 HLA-C1 may increase the risk of CMV infection after kidney transplantation [246]. Finally, Wu et al. indicated that the heterozygote consisting of KIR2DS4 and KIR1D is an independent protective factor against CMV reactivation after HSCT [247].

Overall, the literature highlights the importance of considering KIR genetic variability to improve the control and management of CMV infection, especially in transplant recipients and immunocompromised individuals.

### 6.2. Ebola Virus Disease

Ebola virus disease (EVD) is a severe acute viral illness that can lead to severe and often fatal hemorrhagic fever in humans and other primates. It is caused by the Ebola virus (EV), an RNA virus of the Filoviridae family, transmitted through direct contact with infected blood, bodily fluids, or organs. Episodes of EVD have been predominantly limited to localized outbreaks in Central and West Africa. Initial symptoms may include sudden fever, headache, sore throat, intense muscle pain, weakness, and fatigue. As the disease progresses, more severe symptoms can occur, including vomiting, diarrhea, rash, compromised kidney and liver function, and, in some cases, internal and external bleeding.

The genetic variability of KIR receptors could influence the immune response and the severity of EVD. In 2010, Wauquier et al. analyzed the correlation between KIR genes and EVD, finding that the activating KIR2DS1 and KIR2DS3 genes are associated with fatal outcomes in EV infection [248]. These results support the hypothesis that excessive immune response activation is responsible for the rapid depletion of NK cells and lymphocytes. However, a subsequent study by Wawina-Bokalanga and colleagues found that activating KIR2DS1 and KIR2DS3 genes did not significantly correlate with disease outcomes [249]. Additionally, the authors observed that the KIR haplotype lacking the genes KIR2DL2, KIR2DL5, KIR2DS1, KIR2DS2, KIR2DS3, KIR2DS5, and KIR3DS1 was significantly more common among individuals who died of EVD compared to survivors. KIR2DS4-003 and KIR2DL5 were significantly more frequent in individuals who died from EVD than survivors. These results highlight that EVD survivors express fewer activating and more inhibitory KIR genes, and more functional inhibitory KIR/HLA pairs compared to individuals who died from EVD.

Further research is needed to fully understand how these receptors influence the host response to the Ebola virus and to develop targeted therapeutic strategies.

### 6.3. Epstein–Barr Virus

The Epstein–Barr virus (EBV) is a herpesvirus associated with various clinical conditions, including infectious mononucleosis, a viral disease characterized by fever, sore throat, swollen lymph nodes, and fatigue. In addition to mononucleosis, EBV has been implicated in some autoimmune diseases and certain types of cancer, such as Hodgkin lymphoma; some types of non-Hodgkin lymphoma; and some forms of nasopharyngeal carcinoma. The virus is primarily transmitted through direct contact with infected saliva and other biological fluids. After the primary infection, the virus remains latent in B lymphocytes and can reactivate under conditions of immunosuppression.

Several studies have explored the association between KIR genes, EBV infection, and related pathologies. In 2015, in patients with EBV and hemophagocytic lymphohistiocytosis, a rare immune system dysfunction, the frequencies of the KIR2DS1, KIR2DS5, KIR3DS1, and KIR2DL5 genes were higher compared to healthy controls. Conversely, patients with infectious mononucleosis had higher KIR2DS2, KIR2DS4, KIR2DL2, and KIR2DL5 frequencies than healthy controls. Additionally, the frequency of KIR3DS1 was higher in patients with EBV and hemophagocytic lymphohistiocytosis compared to those with mononucleosis, suggesting that KIR3DS1 might be a risk factor in the progression of EBV infection toward hemophagocytic lymphohistiocytosis [250].

A study on HSCT revealed that donors with activating KIR genes, such as KIR2DS1, KIR2DS3, and KIR3DS1, increased the risk of EBV reactivation in transplanted patients [251]. However, a subsequent study reported that these same KIR genes might instead protect against EBV reactivation in patients with lymphoid diseases after transplantation [252].

The role of KIR in susceptibility to EBV-associated classic Hodgkin Lymphoma has also been explored. Jang et al. showed that individuals carrying KIR haplotype B, especially genes KIR2DL2–HLA-C1 and KIR2DS2–HLA-C1, had significantly lower frequencies among EBV+ cHL patients compared to healthy controls, suggesting a protective role of KIR haplotype B against this disease [253].

Finally, a study on the Kenyan population showed that having at least four activating KIR genes predisposes individuals to endemic Burkitt’s lymphoma. Patients with endemic Burkitt’s lymphoma had high levels of EBV in the Bx haplogroup and AB genotypes compared to healthy controls [254].

These findings highlight the complex interaction between KIR genes and EBV pathogenesis, suggesting that these genes could be potential biomarkers for assessing EBV-related disease risks and guiding therapeutic strategies.

### 6.4. Hepatitis B Virus

HBV is a virus that infects the liver and causes both acute and chronic infections. It is transmitted through contact with infected blood or bodily fluids or from mother to child during childbirth. Hepatitis B is a severe disease that affects approximately 296 million people worldwide and is a leading cause of complications such as cirrhosis and liver cancer [255].

Some studies have highlighted the influence of KIR genes on susceptibility to HBV infection. For example, KIR2DS2 and KIR2DS3 increase susceptibility, while KIR2DS1, KIR3DS1, and KIR2DL5 may confer protection [256]. In 2014, Kibar et al. identified specific KIR genes, such as KIR2DL3 and KIR3DS1, associated with increased resistance to HBV infection [257]. Di Bona et al. observed that the frequency of KIR2DL3 was lower in subjects with chronic hepatitis B compared to those with resolved infection [258]. A protective role of KIR2DL3 was also observed in a study conducted on the Sicilian population. The same study also noted that KIR3DL1 was more frequent in patients with chronic hepatitis B [223]. Finally, Torimiro and colleagues found a higher frequency of KIR3DL1, KIR2DL1, and KIR2DS4 and a lower frequency of KIR2DL2 and KIR3DS1 in patients with HBV compared to healthy subjects [259].

Other genetic variants influence the course of HBV infection. In a population from Burkina Faso, KIR2DL2, KIR2DL3, and KIR2DS2 are associated with chronic stages of HBV infection, while KIR3DL1, KIR3DL2, KIR2DS1, and KIR2DP1 are associated with immunity against the infection [260]. In subsequent studies, it was reported that KIR2DS4 increases the risk of chronic infection, KIR2DL2 and KIR2DL3 are associated with a lower risk, and KIR2DS5 and the KIR2DP1 pseudogene are linked to susceptibility to occult HBV infection [261,262]. KIR2DL3 is also associated with protection against occult HBV infection [262]. Additionally, Yindom et al. reported that subjects with KIR3DS1 are more likely to be positive for the HBV antigen e (HBeAg), a marker of persistent replication, and have a high viral load [263].

The impact of KIR genes on virus elimination has also been investigated. Patients recovered from the virus have a higher frequency of KIR2DL5A, KIR2DS1, and KIR3DS1. KIR2DL5 and KIR3DP1, activating phenotypes, are associated with virus elimination in infected individuals [264].

Some genetic variants also influence the response to therapy. A higher frequency of KIR3DS1 has been observed in patients with a sustained response to IFN-α treatment [265]. Zhuang et al. reported that patients with the KIR2DS3 gene might respond negatively to anti-HBV therapy with Entecavir. In contrast, carriers of the KIR2DL3 and KIR3DS1 genes might benefit more from the therapy [266].

This evidence is crucial for understanding hepatitis B pathogenesis and developing personalized therapeutic approaches considering individual genetic variability.

### 6.5. Hepatitis C Virus

HCV is a virus that causes acute and chronic hepatitis. Infection primarily occurs through contact with infected blood or bodily fluids. HCV is known for persisting in the liver for long periods, often without causing clinical symptoms. This can lead to a chronic infection that progressively damages the liver over time. Individuals with chronic HCV infection are at risk of developing serious complications such as cirrhosis and HCC, which may require complex treatments like liver transplantation.

In recent years, studies have highlighted associations between HCV and KIR genes, which could offer new insights into the pathogenesis of hepatitis C and the development of new therapeutic strategies. In 2004, it was demonstrated that the KIR2DL3 and HLA-C1 genes influence the resolution of HCV infection in Caucasians and African Americans exposed to low doses of the virus but not in those exposed to high doses [267]. The protective role of KIR2DL3 has been highlighted in various clinical profiles following exposure to HCV virus, confirming its positive impact on different subgroups: those who show resistance to HCV infection once contracted, those who resolve the infection spontaneously, and those who respond to chronic treatment with IFN-α [268]. A study on a Caucasian population in Latin America revealed that patients who clear the virus exhibit a decrease in KIR2DL2 and KIR2DS2 and an increase in KIR2DS5. In contrast, those who fail to clear the virus show increased activation of KIR receptors [269]. However, other studies have not identified any significant association between KIR genes and susceptibility to HCV infection, progression to fibrosis or cirrhosis, or resolution of infection [270,271].

A study conducted on a group of intravenous drug users not infected but exposed to HCV suggests a protective role against HCV infection for KIR2DL2 and KIR2DL3 in the presence of KIR2DS4 [272].

A subsequent study associated the KIR2DS5 gene with a sustained virological response (SVR) and reported an association between the KIR2DL2, KIR2DS2, and KIR2DS3 genes and chronic infection [273]. The KIR2DS2 and KIR2DL2 genes appeared to increase the risk of chronic HCV infection. Additionally, the average viremia levels were lower in individuals having the KIR2DS3 gene but not KIR2DS5 [274].

A recent meta-analysis identified several significant associations between KIR and HLA in patients with HCV infection. KIR2DS3 is correlated with spontaneous and treatment-induced clearance, while the presence of KIR2DL2 and KIR2DS4 shows a trend toward lower SVR rates after IFN-based therapy [275]. Regarding mother-to-child transmission, KIR2DS1 and KIR3DS1 favor viral persistence in the mother, while KIR2DS3 favors viral clearance in children [276].

KIR and HLA genotyping showed no differences between patients with chronic infection and controls. Noteworthy individuals with high viral loads showed an increased expression of KIR2DS3 and KIR2DS4. Thus, the authors concluded that homozygosity for KIR2DS4 is associated with liver cirrhosis. Additionally, in individuals with a shorter duration of infection who progressed to cirrhosis, a reduction in the expression of KIR3DL1 was observed [277].

Studies on the Chinese population have shown that the KIR2DL5A-/KIR2DL5B+ genotype is correlated with spontaneous clearance of HCV, while the KIR2DL3/KIR2DL3 and KIR2DL3/KIR2DL3+HLA-C1 genotypes are associated with chronic infection [278]. Genetic variants of KIR2DS4, KIR2DS1, KIR2DL1, and HLA-C increase susceptibility to infection [279]. Specific polymorphisms of KIR2DL4/HLA-G are associated with susceptibility to infection in high-risk populations. These genes could influence the immune system response to HCV infection [280]. Finally, Li et al. observed a significant association between variations in the KIR3DL2/HLA-A gene and an increased susceptibility to HCV infection [281].

In conclusion, current evidence provides important insights into the pathogenesis of hepatitis C. However, variations in the studied populations indicate that further research is necessary to elucidate these mechanisms fully.

### 6.6. Herpes Simplex Virus

The herpes simplex virus (HSV) causes skin lesions such as cold sores and genital herpes. It spreads through direct contact with infected lesions or body secretions of infected individuals, even in the absence of apparent symptoms. After the initial infection, it can remain latent in the body and reactivate periodically, causing recurrent skin eruptions. Additionally, HSV can lead to serious complications such as encephalitis, keratitis, and more widespread systemic infections in immunocompromised individuals.

Several studies have examined a potential correlation between KIR genes and HSV infection. The KIR2DL2 and KIR2DS2 receptors appear to play a key role in the susceptibility and severity of the infection, particularly when KIR2DL2 is combined with HLA-C [282,283]. Moreover, the absence of KIR2DS4del and specific HLA-Bw4 alleles may exacerbate cutaneous lesions [284]. Finally, an analysis found that specific KIR haplotypes, such as the AA haplotype and HLA variants, are associated with a higher incidence of HSV-related viral encephalitis [285].

In conclusion, these studies suggest that genetic variability in KIR genes can influence the incidence and severity of cutaneous manifestations and systemic complications of HSV. Understanding these genetic interactions could open the way for personalized therapeutic strategies to improve HSV infection control and prevent serious clinical consequences.

### 6.7. Human Herpesvirus 8

Human Herpesvirus 8 (HHV-8) is associated with Kaposi’s sarcoma (KS), a vascular neoplasm affecting the skin, other internal organs, and other lymphoproliferative disorders. HHV-8 is primarily transmitted through contact with infected secretions or via organ transplantation. The virus can remain latent in the body for long periods without causing symptoms but can reactivate in immunocompromised individuals. Several studies have highlighted how specific KIR genotypes are associated with the susceptibility and severity of HHV-8 infections and the development of KS.

A study found that activating genotypes such as KIR3DS1, KIR2DS1, and KIR2DS1 in combination with HLA-C1 are more frequent in KS than controls [286]. Patients with HHV8 infection show a higher frequency of homozygosity for KIR2DL2/KIR2DS2 and a concomitant decrease in homozygosity for KIR2DL3, and this combination significantly increases the risk of skin lesions compared to individual factors [287]. HLA-C1 alleles, which act as ligands for the inhibitory receptors KIR2DL2 and KIR2DL3, protect against HHV-8 seroprevalence but increase the risk of KS in infected individuals. KIR2DL2 increases the risk of developing KS in HHV-8-positive immunocompetent individuals, according to Bartolotti’s study [288]. The combination of KIR3DS1 and HLA-Bw4-80I protects against HHV-8 seroprevalence but is associated with an increased risk of developing KS [289]. Finally, increased KIR2DL3 has been correlated with a high viral load in HHV-8 seropositive individuals. Characterizing the KIR genotype in these individuals revealed variations in the frequency of KIR2DL2 and KIR2DL3 genes [290].

Further studies are needed to better understand the interactions between KIR genes and HHV-8 and to develop targeted therapeutic strategies to improve the management and prevention of these diseases.

### 6.8. Human Immunodeficiency Virus

The human immunodeficiency virus (HIV) compromises the immune system by damaging CD4+ T lymphocytes and, if untreated, leads to acquired immunodeficiency syndrome (AIDS). The virus is primarily transmitted through contact with infected blood; unprotected sexual intercourse with an infected person; or from mother to child during pregnancy, childbirth, or breastfeeding. HIV infection is characterized by three stages: acute phase, clinical latency phase, and AIDS. The acute phase is characterized by flu-like symptoms within 2–4 weeks of initial infection. The clinical latency phase is an asymptomatic period or a period with mild symptoms that can last many years. AIDS is the advanced stage of HIV infection, characterized by severe immune system compromise and opportunistic infections. HIV infection represents one of the main areas of interest in the role of KIR genes.

The activating allele KIR3DS1, combined with specific HLA-B alleles, slows the progression to AIDS, whereas KIR3DS1 without these alleles is associated with a faster progression [291]. Moreover, having two copies of KIR3DS1 promotes more vigorous activity of NK cells, increasing resistance to HIV [292]. Combinations of alleles at the HLA-B and KIR3DL1 loci can protect against disease progression and viral load [293,294,295], also showing a protective effect against AIDS [296,297]. The copy number variation region involving the KIR3DS1 and KIR3DL1 genes influences the control of HIV-1. An increase in copies of KIR3DL1 protects against HIV only in the presence of at least one copy of KIR3DS1, with an effect correlated to the increase in KIR3DS1+NK cells and their ability to suppress the virus [298]. Furthermore, NK cells expressing KIR3DS1 and, to a lesser extent, KIR3DL1 specifically expand during acute HIV-1 infection in the presence of HLA-B Bw480I, the putative class I HLA ligand for these receptors [299].

KIR2DS2 is associated with a more rapid decline in CD4+ T lymphocytes and progression to AIDS without affecting viral load [300]. The presence of inhibitory KIR genes without their respective HLA genes protects women exposed to the virus, resulting in seronegativity. Conversely, seropositive women often show corresponding pairs of inhibitory KIR genes with HLA ligands. Consequently, NK cells can be more easily activated if the binding for inhibitory KIR genes is missing. Moreover, AB KIR genotypes, which include a more significant number of activating KIRs, are associated with more excellent resistance to infection [301]. KIR group B haplotypes and the absence of genes specifically binding to inhibitory KIRs are associated with a reduction in CD4+ T lymphocytes during HIV infection [302]. Jennes et al. found that specific mismatched KIR/HLA combinations are associated with transmission or absence of HIV, suggesting that non-reactive NK cells play a key role in protective immunity against the virus [303].

A study conducted on the Polish population highlighted KIR2DL3 as a protective factor, with a similar effect for KIR3DS1 in intravenous drug users. However, KIR2DL5 is associated with an increased risk of HIV infection, along with KIR2DL2 in women and KIR2DS1 in intravenous drug users [304].

A meta-analysis showed that KIR2DS4 is associated with an increased risk of HIV infection, while KIR3DS1 reduces the risk. Among Africans, KIR2DS4 confers a significant risk of infection, while KIR2DL2, KIR2DL5, and KIR2DS3 provide protection. Among Caucasians, KIR2DL2 and KIR3DL1 are associated with increased risk, while East Asians show a negative association between KIR2DL1, KIR2DL3, and KIR3DS1 and HIV infection. KIR3DS1 has shown a protective effect in serodiscordant couples. Lastly, the reduced frequency of KIR2DL3 in Chinese individuals appears to be correlated with a slower disease progression [305]. A study conducted in Burkina Faso reported that the KIR2DL2, KIR2DS2, KIR2DS3, KIR2DS4, and KIR3DS1 genes were associated with HIV-1 infection, while the KIR3DL1 gene was associated with protection against HIV-1 infection [306].

KIR genes also contribute to susceptibility to and protection against mother-to-child viral transmission. An investigation conducted on mothers and newborns in sub-Saharan Africa found that the KIR2DL2/KIR2DL3 genotype was less frequent in mothers transmitting during childbirth. At the same time, homozygosity for KIR2DL3, especially in combination with HLA-C1C2 heterozygosity, was higher in transmitting mothers. Furthermore, in newborns, the presence of KIR2DL3 in combination with the HLA-C1 ligand and homozygosity for KIR2DL3 with HLA-C1C2 were both less common in infected newborns, suggesting a protective role [307]. A subsequent study conducted in Cameroon showed that the frequency of KIR2DL1 was significantly higher in the HIV-unexposed group compared to the exposed group and that some KIRs, such as KIR2DS1, KIR2DS5, and KIR2DL5, were more present in uninfected newborns [308].

Further studies have been conducted regarding the effects of KIR genes on the immune response to combination antiretroviral therapy (cART) in HIV. It has been observed that the presence of the KIR2DL3 allele has a strong protective effect against becoming an immunological non-responder [309].

The diversity in KIR genetic profiles among different populations underscores the complex interaction between host genetics and immune response in HIV pathogenesis. Further research is crucial to fully understanding these mechanisms, developing new approaches to enhance the clinical management of affected patients, and refining therapeutic strategies.

### 6.9. Human Papillomavirus

HPV is a virus that infects human skin and mucous membranes, primarily transmitted through direct contact with infected skin or mucous membranes. There are various types of HPV, classified into “low-risk” and “high-risk” based on the risk of developing precancerous and cancerous lesions. Low-risk HPV can cause genital warts, while high-risk HPV is associated with precancerous and particularly CN lesions. Many HPV infections are asymptomatic, and the immune system can spontaneously clear the virus within months or years. However, persistent infections with high-risk HPV can progress to precancerous lesions and subsequently to cancer. The KIR genes can influence the immune response against HPV, potentially modulating the risk of developing precancerous and cancerous conditions associated with the virus.

A study indicated the potential correlation between KIR genes and recurrent respiratory papillomatosis (RRP), a disease caused by HPV infection characterized by the recurrent formation of benign tumors in the larynx and upper airways. Patients with HPV-6/11 lacking both KIR3DS1 and KIR2DS1 genes seem more prone to developing severe RRP, suggesting that NK cells expressing these activating receptors may play a crucial role in triggering an effective immune response against the virus. Additionally, a decrease in the inhibitory receptor–ligand pair KIR3DL2+HLA-A3/11 was observed in RRP patients, suggesting a potential protective effect of this interaction against the development of the condition [310].

Regarding cervical intraepithelial neoplasia, studies conducted in various populations have not found any significant association [157,311,312,313,314]. However, an investigation in an Indian population showed a significant association between the KIR2DS5 gene and cervical carcinoma, suggesting a susceptibility role, while KIR2DL1 and KIR2DL5B have a protective role. Additionally, a significantly higher frequency of KIR2DL3 was observed in women with HPV infection compared to those with cervical carcinoma, suggesting a possible protective effect against tumor development [158]. This latter evidence contrasts with a study on an Italian cohort, which found an association between the KIR2DL2/HLA-C1 and KIR2DL3/HLA-C1 pairs and invasive cervical cancer at high risk [315].

The variability of the results underscores the importance of further research to better understand the role of KIR genes in the progression of HPV infections and related diseases.

### 6.10. Leprosy

Leprosy, or Hansen’s disease, is a severe infectious disease caused by Mycobacterium leprae. The disease primarily affects the skin, peripheral nerves, mucous membranes of the upper respiratory tract, eyes, and other organs. Transmission occurs mainly through prolonged close contact with infected individuals but is not highly contagious. The disease typically has a long incubation period that can last several years and can result in permanent disabilities if not treated properly. Patients with leprosy have immune dysfunction characterized by defective T-cell activation; overexpression of inhibitory pathways, such as PD-1 on Tregs, leading to suppressed immune responses; and increased levels of immunosuppressive cytokines like IL-10, IL-35, and TGF-β, especially in severe cases. Genetic predisposition plays a significant role in susceptibility [316]. KIR genes are involved in the development and severity of leprosy, influencing the patient’s immune response. Franceschi et al. were the first to investigate the role of KIR genes in leprosy subtypes. They found that patients with tuberculoid leprosy, a milder form of the disease, have a higher frequency of KIR2DS3 and KIR2DS2 genes compared to those with lepromatous leprosy, indicating potential protection against more severe forms of the disease [317]. Jarduli et al. observed that activating KIR genes, such as KIR2DS1, KIR2DS2, and KIR3DS1, might confer protection against the more aggressive form of leprosy. They also noted a higher frequency of KIR2DL2 in patients than controls [318]. Lastly, Alves et al. highlighted associations between KIR2DL2 and KIR2DL3 and increased risk of developing leprosy in its more severe clinical or borderline form. Additionally, they found that KIR3DL2 and its ligand act as a risk factor for the borderline form of the disease [319].

This evidence highlights genetics’ importance in understanding the variation in susceptibility to leprosy and potentially improving treatment and prevention strategies.

### 6.11. Leptospirosis

Leptospirosis is an infectious disease caused by bacteria of the genus Leptospira, primarily transmitted through the urine of infected rodents, contaminated water, or contaminated soil. Symptoms of leptospirosis range from mild to severe and can include fever, headache, muscle pains, chills, nausea, vomiting, jaundice, bleeding, and kidney failure. In severe cases, it can progress to Weil’s syndrome, which can lead to potentially fatal complications. Leptospirosis is prevalent in rural and tropical environments.

Studies on KIR in the susceptibility to leptospirosis are limited, and current results are conflicting. A preliminary study in 2006 suggested a possible involvement of the genes KIR2DL3, KIR2DL5B, KIR2DS1, and KIR2DS5 [320]. However, a subsequent study performed in 2009 on a cohort of patients from the Azores islands did not confirm these associations, finding no correlation between KIR genotypes and susceptibility to leptospirosis [321].

Further investigations are needed to understand the role of KIR genes in leptospira infection fully.

### 6.12. Malaria

Malaria is caused by parasites of the genus Plasmodium, transmitted to humans through bites from infected mosquitoes. The disease is endemic in many tropical and subtropical regions. The main symptoms include fever, nausea, vomiting, and muscle pain. In more severe cases, it can cause severe anemia, respiratory failure, and complications in the kidneys and brain, potentially leading to death.

KIR genes have been associated with malaria, influencing both the severity of the disease and the immune response. Malaria and KIR genes have been associated with KIR3DL1/KIR3DS1 and KIR2DS4 genotypes. KIR3DL1 might inhibit the activation of KIR3DS1, thus controlling the parasite proliferation [322]. Additionally, KIR3DL1 has been positively associated with malaria severity [323].

KIR2DL3, associated with HLA-C1, has been linked to the development of cerebral malaria, a potentially lethal complication [324]. Also, KIR2DS2/HLA-C1 and KIR2DL2/HLA-C1 are more frequent in malaria patients compared to healthy subjects, with KIR2DS2, KIR3DS1, and KIR2DS5 associated with slightly higher mortality in Gambia [325].

In the Nigerian population, KIR2DS5 and KIR2DS3 may be protective against severe malaria [326]. In a population from northern India, KIR2DS2, KIR2DL1, and KIR2DL3 were associated with complicated malaria [327]. Finally, in a cohort of Ugandan individuals, it was observed that the presence of the HLA-C2 and HLA-Bw4 alleles, which are ligands for the inhibitory receptors KIR2DL1 and KIR3DL1, respectively, increased the likelihood of Plasmodium falciparum parasitemia. This suggests that KIR-mediated inhibition confers a higher risk of Plasmodium falciparum parasitemia [328]. However, no significant association was found among the northern population [329]. Finally, Tukwasibwe et al. hypothesized that genetic variation in *KIR* may differ in Ugandan populations with historically varied malaria transmission intensity [330]. The authors found that the KIR3DS1, KIR2DL5, KIR2DS5, and KIR2DS1 genes may partly explain differences in malaria transmission intensity since these genes were positively selected for places with historically high malaria transmission intensity.

In summary, the results suggest that associations between KIR genes and malaria can vary significantly among different populations, underscoring the importance of further studies to deepen our understanding of these relationships. This is crucial for developing effective strategies for preventing and treating the disease.

### 6.13. Q Fever

Q fever is a zoonotic infectious disease caused by the bacterium Coxiella burnetii, an obligate intracellular pathogen with a remarkable capacity for infection [331]. The disease is globally prevalent and presents a variety of clinical features and geographical distributions. It is particularly common among individuals living in rural areas who have direct contact with animals. Transmission to humans primarily occurs through inhaling aerosols containing the pathogen, especially those originating from placental products. Wild animals, domestic animals, and ticks are the main vectors of the bacterium.

In a preliminary study, patients affected by Q fever fatigue syndrome (QFS), a debilitating condition that can develop after an acute Q fever infection, were genotyped along with healthy controls [332]. The analysis focused on genetic variants in 15 genes and the frequencies of HLA alleles. The results show a significant increase in the frequency of HLA-DR11 in QFS patients, along with differences in the natural resistance-associated macrophage protein (NRAMP) and IFN-γ genes, suggesting a possible genetic role in developing chronic Q fever. Subsequently, the same research group analyzed the frequencies of immune response gene variants in three patient groups: patients with QFS, patients with Q fever endocarditis (QFE), and patients who recovered without complications after an initial acute Q fever attack [333]. The authors observed that patients with QFS show variations compared to patients with QFE and those without complications and to the controls, particularly in the frequency of carriers of HLA-DRB111. The presence of the HLA-DRB111 allele is associated with reduced responses of IFN-γ and IL-2 from Peripheral Blood Mononuclear Cells (PBMCs).

Currently, no studies have examined the association between KIR genes and Q fever. Several reasons can explain this lack of research. Attention is often directed towards genes that directly impact the immune response, such as those that produce cytokines.

### 6.14. Sepsis

Sepsis is a clinical condition characterized by life-threatening organ dysfunction caused by a dysregulated host response to infection. It represents a major challenge in critical care medicine due to its high mortality rates and complex pathophysiology. It typically begins with an infection that triggers a systemic inflammatory response. This response can lead to widespread tissue damage, impaired microcirculation, and ultimately, multiple organ dysfunction syndrome (MODS). Recent research has suggested a genetic component to sepsis susceptibility, particularly involving the KIR genes.

In 2017, Oliveira et al. identified some variants of the KIR genes KIR2DS1 and KIR3DS1 in patients without sepsis compared to patients with sepsis, suggesting that these genes may have a protective role [334]. This finding indicates that genetic variations in the immune response could influence an individual’s susceptibility to sepsis. Moreover, in 2019, the same researchers highlighted that the KIR haplotype A might be associated with a higher likelihood of hospital mortality of 56% in septic patients [335]. This association suggests that certain genetic profiles may predispose individuals to worse outcomes when afflicted with sepsis.

Further studies are needed to clarify the role of KIR genes in determining sepsis susceptibility and to develop therapeutic strategies against sepsis.

### 6.15. Severe Acute Respiratory Syndrome Coronavirus 2

Severe Acute Respiratory Syndrome Coronavirus 2 (SARS-CoV-2) is responsible for the disease known as Coronavirus Disease 19 (COVID-19), which is primarily spread through respiratory droplets. COVID-19 can cause various symptoms, ranging from mild to severe, including fever, cough, difficulty breathing, fatigue, and other flu-like symptoms. In severe cases, it can lead to serious complications and even death, especially in elderly individuals or those with pre-existing medical conditions.

Numerous studies have explored the link between KIR genes and susceptibility to COVID-19. A decrease in circulating NK cells and a prevalence of inhibitory receptors after SARS-CoV-2 infection have been noted, suggesting a weakened NK-cell response [336]. Genetic analysis in a Sardinian population revealed a prevalence of inhibitory KIR receptors like KIR2DL1 and KIR2DL3 in COVID-19 patients, indicating increased susceptibility in individuals with such genetic profiles. Conversely, the KIR2DS2/HLA-C1 combination may protect against adverse disease outcomes [337]. A meta-analysis confirmed that KIR2DL3 is associated with increased COVID-19 risk, while KIR2DP1 may reduce this risk. Specific genes, such as KIR2DS4 and KIR3DL1/KIR3DL2, have been linked to greater disease severity [338]. However, Ligotti et al. did not find significant differences in the distribution of KIR genes and their HLA ligands between asymptomatic/paucisymptomatic individuals and symptomatic COVID-19-positive individuals in a Sicilian population [223].

The KIR2DL1/HLA-C2 complex has been linked to increased disease severity [339], and the frequency of KIR2DS4 and KIR3DL1 genes has been associated with a risk of severe COVID-19, suggesting a significant role of these genetic markers in antiviral immunity [340]. KIR2DS4 has also been associated with COVID-19 in other populations [341,342].

Specific KIR-HLA combinations that control NK-cell function are critical in antiviral immunity, and the absence of certain combinations, such as KIR3DL1/HLA-Bw4 and KIR3DL2/HLA-A3/11, may lead to compromised defense against the virus. Furthermore, an increased frequency of KIR2DS1 and KIR2DS5 in severely affected COVID-19 patients suggests a robust NK-cell response triggered by these activating receptors and subsequent excessive production of inflammatory cytokines responsible for severe disease [343].

Finally, the KIR2DL2 gene has been associated with increased COVID-19 risk, and its frequency has been positively correlated with the number of cases in certain geographical regions [344]. The KIR2DL2/HLA-C2 combination has also been associated with increased susceptibility to the disease [345].

In conclusion, research suggests that KIR genes and their combinations with HLA ligands play a significant role in the immune response to COVID-19. However, further research is needed to understand how these genetic interactions influence the course and outcome of COVID-19, guiding the development of targeted therapies and disease management strategies.

### 6.16. Syphilis

Syphilis is a sexually transmitted disease caused by the bacterium Treponema pallidum. The transmission occurs primarily through direct sexual contact with syphilitic lesions on the skin of the genital, anal, or oral areas. Less commonly, it can be transmitted through infected blood transfusions or from mother to child during pregnancy (congenital syphilis). If left untreated, the disease progresses through various stages characterized by skin rashes, fever, sore throat, swollen lymph nodes, and flu-like symptoms. Later, it can lead to severe neurological and cardiovascular complications or even be fatal.

Currently, there are few studies in the literature that have examined a possible association between KIR genotypes and syphilis. An analysis of KIR genotypes in patients with syphilis and healthy controls from the Chinese Han population revealed that the KIR Tel-B/B genotype is significantly more frequent in patients with syphilis. This genotype is associated with increased activation of KIR genes in the Tel region compared to the Cen region, suggesting that the Tel-B/B genotype, encoding a set of activating KIR genes, contributes to an increased risk of syphilis [346].

Further research examined KIR2DS4 and its variant KIR1D. The frequency of homozygous KIR1D was significantly higher in patients with syphilis compared to controls, suggesting that the KIR1D/KIR1D genotype may increase the risk of syphilis by interfering with the immune response. However, no significant differences were found in the genetic frequencies of KIR2DS4/KIR2DS4 and KIR2DS4/KIR1D between the two groups [347].

Finally, another study found that KIR2DS3 and KIR3DS1 are associated with an increased risk of syphilis, while KIR2DS5 is associated with a reduced risk. Additionally, the KIR2DL3/HLA-C1C1 genotype is more common in healthy controls compared to patients with syphilis, suggesting a possible synergistic protective effect between HLA-C1 and KIR2DL3 [348].

It is important to note that these findings specifically apply to the Chinese Han population, so further research on different ethnicities is necessary to confirm these associations.

### 6.17. Tuberculosis

Tuberculosis (TB) is an infectious disease caused by Mycobacterium tuberculosis, primarily affecting the lungs. It spreads through respiratory droplets containing the bacteria released into the air when an infected person coughs, sneezes, or speaks. Tuberculosis can manifest in two forms: (i) Latent TB (LTB), characterized by bacteria in the body without causing symptoms, is not contagious. However, LTB may develop into active TB (ATB), especially if the immune system is compromised. (ii) ATB can present with persistent cough, fever, night sweats, and weight loss. Without treatment, ATB can lead to severe lung damage and potentially fatal complications.

Associations have been observed between KIR genes and TB. A cross-sectional study found that the KIR2DL3 gene had a higher frequency in individuals with ATB than controls, although this finding was not statistically significant. Conversely, the KIR2DS3 gene was less frequent in individuals with ATB than in controls. KIR2DL5, KIR2DL5B, and KIR2DS2 showed significant differences among groups with ATB and LTB, and controls. Specifically, LTB patients had an increased frequency of inhibitory KIR genes, such as KIR2DL5 and KIR2DL5B. ATB patients had a decreased frequency of activating KIR genes, such as KIR2DS2. In total, 66.7% of individuals with TB had a centromeric AA haplotype characterized by fewer activating genes. These differences in KIR gene frequencies at various stages of the disease suggest a differential cytokine expression, contributing to different disease outcomes, and indicate a genetic influence on TB susceptibility and pathogenesis [349]. A meta-analysis from 2020 identified a significant association between specific KIR genes and the risk of pulmonary TB infection. Specifically, a positive association was observed between the risk of infection and the following genes: the inhibitory genes KIR2DL3 and KIR3DL1, along with the activating genes KIR2DS1 and KIR2DS4. In contrast, the other KIR genes and the two pseudogenes, KIR2DP1 and KIR3DP1, did not significantly correlate with the risk of pulmonary TB infection. These findings suggest a potential role of KIR genes in determining susceptibility to pulmonary TB infection [350].

**Table 3:** KIR genes and infectious diseases.

	Predisposing KIRs	Protective KIRs
Cytomegalovirus	KIR2DL1 [225] KIR2DL3 [225,226] with HLA-C1 [235,244] KIR3DS1 [225,226,227,240] KIR3DL1 [226] with HLA-Bw4T [229] KIR2DL2 with HLA-C1 [246] KIR2DS2 [229,232,238,244] with HLA-C1 [237] KIR2DS4 [230,238] KIR2DS5 [231,240] KIR2DS2 [231] KIR2DS1 [240] KIR2DL5A [240]	KIR2DS1 [227,230,231,236,239,241,242,243] KIR2DS5 [239,243] KIR2DL5A [239] KIR2DS3 [243] KIR1D [247] KIR2DS2 [229,232,245] KIR2DS4 [231,232,245] KIR3DS1 [239,243]
Ebola Virus Disease	KIR2DS1 [248] KIR2DS3 [248] KIR haplotype without KIR2DL2, KIR2DL5 KIR2DS1, KIR2DS2, KIR2DS3, KIR2DS5, and KIR3DS1 [249] KIR2DS4-003 [249] KIR2DL5 [249]	/
Epstein–Barr Virus	KIR2DS1 [250,251] KIR2DS5 [250] KIR3DS1 [250,251] KIR2DL5 [250] KIR2DS2 [250] KIR2DS4 [250] KIR2DL2 [250] KIR2DS3 [251]	KIR2DS1 [243] KIR2DS3 [243] KIR3DS1 [243]
Hepatitis B Virus	KIR2DS2 [256,260] KIR2DS3 [256,266] KIR3DL1 [227,259] KIR2DL1 [259] KIR2DL2 [260] KIR2DL3 [260] KIR2DS4 [259,261] KIR2DS5 [261,262] KIR2DP1 [261,262] KIR3DS1 [263]	KIR2DS1 [256,260,264] KIR3DS1 [256,257,258,264,265,266] KIR2DL5 [256] KIR2DL3 [257,258,261,262,266] KIR2DL2 [259,261] KIR3DL1 [260] KIR3DL2 [260] KIR2DP1 [260] KIR2DL5A [264] KIR2DL5 [264] KIR3DP1 [264]
Hepatitis C Virus	KIR2DL2 [269,273] KIR2DS2 [269,273] KIR2DS3 [273,277] KIR2DS5 [274] KIR2DS4 [277] KIR2DL3/KIR2DL3 [278] KIR2DL3/KIR2DL3 with HLA-C1 [278] KIR2DS1 [276] KIR3DS1 [276] KIR2DS4/KIR2DS1/KIR2DL1 with HLA-C [279] KIR2DL4 with HLA-G [280] KIR3DL2 with HLA-A [281]	KIR2DL3 [268] with HLA-C1 [267] KIR2DS5 [269] KIR2DL2/KIR2DL3/KIR2DS4 [272] KIR2DS5 [273] KIR2DS3 [274,275,276] KIR2DL2 [275] KIR2DS4 [275] KIR3DL1 [277] KIR2DL5A-/KIR2DL5B+ [278]
Herpes Simplex Virus	KIR2DL2 with HLA-C [282,283] KIR2DS2 [282,283]	KIR2DS4del with HLA-Bw4 [284]
Human Herpesvirus 8	KIR2DS1 [286] with HLA-C [286] KIR3DS1 [286] KIR2DL2/KIR2DS2 [287] KIR2DL3 with HLA-C1 [287] KIR2DL2 [277] with HLA-C1 [287] KIR3DS1 with HLA-Bw4-80I [289] KIR2DL3 [290]	KIR2DL3 [287,289] with HLA-C1 [287] KIR2DL2 [289] with HLA-C1 [287] KIR3DS1 with HLA-Bw4-80I [287]
Human Immunodeficiency Virus	KIR3DS1 [291,306,308] KIR2DS2 [300,306] KIR3DS1 [291] KIR2DL5 [304,305] KIR2DL2 [304,305,306] KIR2DS1 [304] KIR2DS4 [305,306] KIR3DL1 [305] KIR2DL3 [305] KIR2DS3 [306] KIR2DL3 with HLA-C1C2 [307]	KIR3DS1 [292,294,305] with HLA-B Bw480I [291,299] KIR3DL1 [306] with HLA-B [291,292,293,294,295,296] KIR3DS1/KIR3DL1 [298] KIR2DL3 with HLA-C1 [307] KIR2DL3 with HLA-C1C2 [307] KIR2DL3 [304,305,309] KIR2DL2 [307,309] KIR2DL5 [305,308] KIR2DS3 [305] KIR2DL1 [305,308] KIR2DL2/KIR2DL3 [307] KIR2DS1 [208] KIR2DS5 [208]
Human Papillomavirus	KIR2DS5 [140] KIR2DL2 with HLA-C1 [315] KIR2DL3 with HLA-C1 [315]	KIR3DS1 [310] KIR2DS1 [310] KIR3DL2 with HLA-A3/11 [310] KIR2DL1 [158] KIR2DL5B [158] KIR2DL3 [158]
Leprosy	KIR2DL2 [317,318] KIR2DL3 [318] KIR3DL2 with its ligand [318]	KIR2DS3 [317] KIR2DS2 [317,318] KIR2DS1 [318] KIR3DS1 [318]
Leptospirosis	KIR2DL3 [320] KIR2DL5B [320] KIR2DS1 [320] KIR2DS5 [320]	/
Malaria	KIR3DL1 [322,324] with HLA-Bw4 [329] KIR3DS1 [322,326] KIR2DS4 [322] KIR2DL3 [328] with HLA-C1 [325] KIR2DS2 [326,328] with HLA-C1 [326] KIR2DL2 with HLA-C1 [326] KIR2DS5 [326] KIR2DL1 [328] with HLA-C2 [329]	KIR2DS5 [327] KIR2DS3 [327]
Q fever	/	/
Sepsis	/	KIR2DS1 [335] KIR3DS1 [335]
Severe Acute Respiratory Syndrome Coronavirus 2	KIR2DL1 [338] with HLA-C2 [338] KIR2DL3 [338,339] KIR2DS4 [338,341,342,343] KIR3DL1/KIR3DL2 [339] KIR3DL1 [342] KIR2DL2 [345] with HLA-C2 [346]	KIR2DS2 with HLA-C1 [338] KIR2DP1 [339] KIR3DL1 with HLA-Bw4 [344] KIR3DL2 with HLA-A3/11 [344] KIR2DS1 [344] KIR2DS5 [344]
Syphilis	KIR1D/KIR1D [347] KIR2DS3 [348] KIR3DS1 [348]	KIR2DS5 [348] KIR2DL3 with HLA-C1C1 [348]
Tuberculosis	KIR2DL5 [349] KIR2DL5B [349] KIR2DS2 [349] KIR2DL3 [349] KIR3DL1 [349] KIR2DS1 [349] KIR2DS4 [349]	KIR2DS3 [349]
Yersinia pestis	/	/
West Nile Virus	/	/
Zika Virus	/	/

### 6.18. Yersinia Pestis

The bacterium Yersinia pestis is the pathogen responsible for the plague, which manifests in three primary forms in humans: bubonic, septicemic, and pneumonic [351]. The most common form is bubonic plague. Rodents are the natural carriers of the bacterium, while infected fleas act as vectors, transmitting the disease to humans through their bites. Transmission also occurs through direct contact and inhalation of contaminated droplets in the case of pneumonic plague. The study by Immel et al. analyzed the DNA of people who died from the plague in the 16th century in Ellwangen, Germany, comparing their HLA genes with those of modern individuals from Ellwangen [352,353]. The authors observed a decrease in HLA-B*51:01 and HLA-C*06:02 in modern populations compared to medieval ones, hypothesizing that these alleles might have been associated with a higher susceptibility to the plague. Conversely, they observed an increase in the frequency of the HLA-DRB1* 13:01/13:02 allele in modern populations, suggesting that it conferred some protection against the disease. These changes in HLA allele frequencies could result from natural selection during plague epidemics, with a potential role of specific alleles in protection or susceptibility to the disease.

However, there are no studies on the association between KIR genes and Y. pestis infection. This lack of research can be attributed to several factors. First, the interaction between KIR genes and their HLA ligands is complex, making it difficult to study their role in rare diseases like the plague. Additionally, research on bacterial infections focused on other immune system components, such as HLA genes, which directly influence the response to pathogens. Finally, studies on the plague are based on historical data and ancient samples. For this reason, it is difficult to understand the role of KIR in the response to Y. pestis infection.

### 6.19. West Nile Virus

The West Nile virus (WNV) is an arbovirus transmitted by mosquitoes. The virus is known to cause a disease called West Nile fever. In severe cases, WNV can lead to encephalitis or meningitis, with symptoms such as fever, headache, fatigue, and severe neurological complications.

A study from 2013 assessed the association between KIR genes and susceptibility to WNV infection. The authors found significant differences between cases and controls for the KIR2DL2, KIR2DS1, KIR2DS2, KIR2DS5, and KIR3DS1 genes. However, it should be noted that the small number of individuals analyzed represents a significant limitation of the study [354].

### 6.20. Zika Virus

The Zika virus (ZIKV) is a flavivirus that causes Zika fever. Transmission occurs primarily through bites from infected mosquitoes, but it can also occur vertically from mother to child during pregnancy, through contaminated blood transfusions, and via sexual contact. The disease can present with mild symptoms such as fever, rash, conjunctivitis, and joint and muscle pain. Still, it can also cause more serious neurological complications, such as Guillain–Barré syndrome in adults, and severe brain abnormalities in newborns, such as microcephaly, when the mother is infected during pregnancy.

A single study has investigated the possible role of KIR genes in ZIKV infection. However, no significant differences were found between cases and controls [355].

## 7. KIR Genes and Neurological Diseases

Neurological diseases encompass many disorders that affect the central nervous system (CNS), peripheral nervous system (PNS), or both. These conditions can impact the brain, spinal cord, nerves, and muscles, leading to diverse symptoms, including motor dysfunction, sensory disturbances, cognitive impairments, and emotional dysregulation.

Neurological diseases vary in their etiology, with causes ranging from genetic mutations and autoimmune disorders to infections, injuries, and environmental factors.

The involvement of KIR in neurological diseases has emerged as an area of growing interest, given the complex interplay between the immune and central nervous systems. The associations between KIR genes and various neurological diseases, such as autism spectrum disorders (ASDs), Parkinson’s disease (PD), and schizophrenia (SCZ), have been examined in several studies (Table 4).

**Table 4:** KIR genes and neurological disease.

	Predisposing KIRs	Protective KIRs
Autism spectrum disorders	KIR2DS5 [356] KIR3DS1 [356,357] KIR2DS1 [356,357] KIR2DS4 [356] KIR2DS2 [357,358]	/
Parkinson’s disease	/	KIR3DL1*015 with HLA-Bw4 [359] KIR3DL1*002 with HLA-Bw4 [359]
Schizophrenia	/	/

### 7.1. Autism Spectrum Disorder

The term autism spectrum disorder (ASD) refers to a group of neurological developmental disorders characterized by deficits in communication and social skills, along with repetitive and stereotyped behaviors. ASD generally manifests in early childhood and can significantly impact learning abilities and daily skills. It is considered a developmental disorder; the exact causes are not yet fully understood but are believed to result from complex interactions between genetic and environmental factors [356].

Several studies have suggested a possible correlation between KIR genes and ASD. The analysis of KIR gene frequencies revealed that some activating genes are common variants with a frequency in ASD above 5% [357]. Torres et al. found an increase in the frequencies of activating KIR genes such as KIR2DS5, KIR3DS1, KIR2DS1, and KIR2DS4 in patients with ASD [356]. Guerini and colleagues examined the distribution of KIR and HLA molecules in patients with ASD and their mothers, finding a predominance of activating KIR/HLA complexes in both groups, particularly KIR2DS2 [358]. The analysis of gene frequencies suggests that three activating genes (KIR3DS1, KIR2DS1, and KIR2DS2) are common predisposing variants. Additionally, analysis of all activating KIR genes revealed an increase in ASD compared to controls [357].

These results suggest that activating KIR genes may be predisposing variants for ASD. However, further studies are necessary to gain a deeper understanding of the genetic factors involved in the development of the disorder.

### 7.2. Parkinson’s Disease

Few studies have analyzed the possible role of KIR genes in the pathogenesis of PD. Anderson et al. analyzed the association of KIR genes with PD as predictors of specific clinical symptoms. They observed that high expression of KIR3DL1 has a protective effect on PD. In particular, the high-expression allele KIR3DL1*015, in combination with HLA-Bw4, is associated with protection from rigidity. Additionally, the high expression of allele KIR3DL1*002, combined with HLA-Bw4, appears protective against gait impairment [359]. These results, therefore, suggest an impact of these NK-cell receptors on the progression of the disease.

These findings suggest a potential impact of KIR genes on the progression of PD. Further research will be crucial to understand these interactions better and develop new therapeutic strategies.

### 7.3. Schizophrenia

Schizophrenia (SCZ) is a chronic psychiatric disorder characterized by psychosis, hallucinations, delusions, abnormal thinking and behavior, as well as reduced emotional expression, motivation, and cognitive functions, resulting in disturbances in daily functioning, including work, social, and self-sufficiency. Although the etiology and pathophysiology of SCZ have not yet been definitively established, studies have shown an increase in the activity of NK cells in patients with this condition, suggesting a potential contribution of these cells to its development [360]. Only one study examined the association between KIR genes and SCZ. In this case-control study, no significant differences were found in the frequencies of KIR genes between 200 patients with schizophrenia and 561 healthy control individuals, nor in the distribution of KIR gene ligands [361].

Further studies are needed to deepen our understanding of the pathophysiology of schizophrenia, mainly to clarify the role of KIR genes and to enhance diagnostic and therapeutic strategies for this condition.

## 8. KIR Genes in Other Diseases

### 8.1. Acute Ischemic Stroke

Acute ischemic stroke (AIS) is a vascular event occurring in the brain due to altered blood flow to a region of the brain caused by an occlusion or spasm of a central vessel. The pathogenesis of AIS involves a complex interplay of factors, including thrombosis, embolism, and systemic hypoperfusion. Inflammation plays a crucial role in the development and progression of AIS. Genetic predisposition also contributes to AIS susceptibility, with specific genetic variants influencing the risk and severity of stroke [362].

Tuttolomondo et al. performed a study to assess whether genetic variants of KIR could influence susceptibility to AIS. It was observed that patients with AIS have a high frequency of the KIR2DL3, KIR2DL4, KIR2DL5B, KIR2DS2, KIR2DS4, and KIR3DP1 genes compared to controls [363] (Table 5). Further studies are mandatory to confirm the preliminary findings of Tuttolomondo et al.

### 8.2. Birdshot Chorioretinopathy

Birdshot chorioretinopathy (BCR) is a rare inflammatory eye condition characterized by bilateral posterior uveitis affecting the choroid and retina. BCR typically presents with symptoms such as floaters, blurred vision, and night blindness and can lead to significant visual impairment if untreated. The pathogenesis of BCR involves an autoimmune response, where the immune system targets retinal and choroidal tissues. The exact mechanisms are not fully understood, but the strong association with HLA-A29 suggests a genetic predisposition. The HLA-A29 allele may present specific T-cell antigens, triggering an autoimmune response. This response leads to chronic inflammation, resulting in the characteristic lesions and retinal degeneration observed in BCR [364].

Only one study identified a significant correlation between specific combinations of KIR genes and HLA loci and the risk of developing BCR. The study revealed that specific allelic combinations of seven KIR and three HLA loci are associated with an increased risk of BCR development. The predominance of specific KIR genes, such as KIR2DS2, KIR2DS3, and KIR2DS4, in BCR patients compared to controls suggests their potential role in enhancing T-cell autoreactivity. Additionally, strong inhibitory combinations of KIR3DL1/HLA-Bw4I80 and KIR2DL1/HLA-C2 in HLA-A*29-negative controls may protect against BCR. These findings underscore the complex interplay between genetic factors and immune responses in BCR pathogenesis [365] (Table 5). Further research is needed to elucidate the pathogenic mechanisms fully. Understanding these genetic influences is essential to improve outcomes for affected patients.

### 8.3. Endometriosis

Endometriosis is a chronic gynecological condition with unknown etiology characterized by an extra-uterine growth of the endometrium. This ectopic endometrial tissue can lead to chronic pelvic pain, dysmenorrhea, and infertility. Immune system abnormalities play a significant role in the pathogenesis of endometriosis. In women with endometriosis, the immune system fails to adequately clear the ectopic endometrial cells, leading to chronic inflammation, which promotes the growth and maintenance of endometriotic lesions [366].

A few studies have found a correlation between KIR genes and endometriosis (Table 5). A lower frequency of KIR3DS1 was observed in patients with endometriosis than in controls [367]. Conversely, endometriosis patients exhibited a protective effect associated with the KIR2DS5 gene, particularly among those with the HLA-C C2 group [368]. In addition, a subsequent study identified a low frequency of KIR2DS2 in patients with endometriosis compared to control groups [369]. These results suggest that KIR genes and their ligands may affect endometriosis. Further investigations into endometriosis’ genetic and molecular bases are essential to explore how KIR gene interactions with their ligands influence the immune environment in endometriosis.

### 8.4. Familial Mediterranean Fever

Familial Mediterranean Fever (FMF) is a chronic inflammatory autosomal recessive disease that mainly affects populations of Mediterranean origin, characterized by recurrent fever, arthritis, peritonitis, pleurisy, skin rashes, and potentially renal amyloidosis. It is primarily caused by mutations in the MEFV gene, which encodes the protein pyrin, which regulates neutrophils during the inflammatory response [370].

The first study on the association between KIR genes and FMF patients reported that the only statistically significant difference between FMF patients and healthy controls in the distribution of activating and inhibitory genes was found in a pseudogene, KIR3DP1*003. This finding suggests a potential role of this pseudogene in FMF, although its biological implications remain unclear and require further research [371] (Table 5). Subsequently, a study by Erken E. et al. found a higher frequency of the KIR2DS2 gene, an activator gene for NK cells, in FMF patients compared to healthy controls [372]. This result highlights the potential involvement of NK-cell activity in the pathogenesis of FMF, suggesting that genetic variations in KIR genes may influence susceptibility and disease severity.

### 8.5. Gaucher Disease

Gaucher disease (GD) is a genetic metabolic disorder caused by glucocerebrosidase (GBA1) gene mutations. This enzyme is responsible for breaking down the beta-glucosidic bond in glucocerebroside lipids. When the enzyme does not function correctly, lipids build up in various tissues and organs in the body [373]. GD is linked to chronic immune stimulation, which can cause inflammation and cell damage through cytokines like IL-1, IL-6, IL-10, and TNF-α, as well as activating NK cells. More evidence of an inflammatory response in GD patients comes from high-antigen-presenting molecules, including MHC class II antigens [374]. GD is associated with a reduction in NK cells [375]. KIR and HLA gene variants might influence the disease’s characteristics. One study explored the relationship between KIR genes and GD in 31 Brazilian patients diagnosed with a biochemical and molecular disease. No significant differences were found when comparing the frequencies of the 15 known KIR genes between GD patients and controls. This was likely due to the small sample size and insufficient statistical power. However, it was noted that the combination of KIR2DS2 and KIR2DL2/HLA-C1 seems to be associated with a delayed onset of symptoms, as it was present in only 15.8% of patients whose symptoms appeared before the age of 18 [376] (Table 5).

It is important to note that the combination of KIR2DS2 and KIR2DL2/HLA-C1 has been described as a protective factor against the development of CML [42]. Indeed, patients with GD are more likely to develop CML [377]. These findings suggest a potential link between KIR and HLA genes and GD, indicating that specific combinations of these genetic variants could influence the onset and progression of the disease.

### 8.6. Paroxysmal Nocturnal Hemoglobinuria

Paroxysmal nocturnal hemoglobinuria (PNH) is a rare acquired genetic disease characterized by nocturnal hemolysis. This condition is caused by a mutation in the PIGA gene in hematopoietic stem cells, resulting in a deficiency of cell membrane proteins that protect red blood cells from attack by the immune system. PNH pathogenesis involves the loss of GPI-anchored proteins like CD55 and CD59, leaving red blood cells vulnerable to complement-mediated lysis. This dysregulation leads to hemolysis, hemoglobinemia, hemoglobinuria, and thrombotic complications due to activated complement components [378].

Only one study investigated the association between KIR and PNH genes, showing that Italian patients with PNH have a reduced expression of activating KIR genes. In addition, inhibitory genotypes, such as KIR3DL1/HLA-Bw4 in patients carrying the HLA-B14:02 and HLA-C08:02 haplotypes, were significantly associated with PNH in Italy [379] (Table 5).

The association between KIR genes, HLA molecules, and PNH highlights the intricate relationship between genetic factors and immune dysregulation in disease development. Further research is crucial to unravel the precise mechanisms underlying these genetic interactions.

### 8.7. Polycystic Ovary Syndrome

Polycystic ovary syndrome (PCOS) is a complex hormonal disorder affecting the endocrine and reproductive systems. PCOS is characterized by hormonal imbalances, particularly involving androgens; insulin resistance; and disrupted ovarian function. While the exact causes of PCOS remain unclear, genetic predisposition, environmental factors, and insulin resistance play significant roles. In recent years, the potential involvement of immune dysregulation has emerged as an area of interest [380].

Only Sala Elpidio et al. explored the potential involvement of KIR genes and their HLA ligands in PCOS immunopathogenesis. The authors found associations between specific KIR genotypes and susceptibility to PCOS. Specifically, KIR3DS1 in interaction with the HLA-Bw4 ligand was found in PCOS patients [381] (Table 5).

### 8.8. Pre-Eclampsia

Pre-eclampsia (PE) is a serious pregnancy-related condition characterized by hypertension and other complications such as proteinuria. It can pose significant health risks for both the mother and the baby, leading to outcomes like preterm birth, perinatal mortality, and increased risk of cardiovascular and metabolic diseases later in life [382].

PE has an immunological basis. The interaction between HLA-C molecules and KIR receptors in maternal cells is key to the condition’s development. Mothers lacking most or all activating KIR (AA genotype), when the fetus possesses HLA-C, have a significantly higher risk of PE, likely due to excessive inhibition of uterine NK cells, compromising trophoblast invasion. When individual KIR genes were analyzed, many were less frequent in mothers with PE, but only the frequency of the KIR2DL5 gene reached statistical significance [383] (Table 5). Subsequently, a study by Li Y et al. demonstrated significantly lower expression of KIR2DL4 in the placenta of women with PE compared to healthy controls [384]. This finding is consistent with Wang D et al., showing that reduced KIR2DL4 levels correlate with susceptibility to PE [385]. In a case-control study, KIR gene frequencies and genotype analyses did not show statistically different results between control groups and patients with PE. Indeed, the ratio of activating to inhibitory genes indicated that inhibitory genes were more common in patients with PE, while activating genes were more common in the healthy control group [386].

In 2014, Yusrizal et al. observed that the expression level of the KIR3DL2 gene is reduced in PE, which could compromise the ability of trophoblasts to protect themselves from maternal immune cell attacks and adequately invade the uterus early in pregnancy. This could lead to poor uteroplacental perfusion and faulty localization [387]. Concurrently, a study demonstrated that the frequency of KIR2DS1 was decreased in Chinese patients with PE compared to healthy controls [388]. At the same time, KIR2DS5 and KIR2DL1 were protective against the disease in African women [389]. Later studies have shown that women with PE have fewer activating KIRs, such as KIR2DS2, KIR2DS3, and KIR2DS5. Additionally, the frequency of the KIR2DL1 gene is increased in women with PE when a homozygous HLA-C2 allele appears in the fetus [390]. This same result has been confirmed by other more recent studies [391,392]. The lack of activating KIRs could reduce the activation of uterine natural killer cells, thus contributing to the pathogenesis of PE [390]. PE represents a significant challenge during pregnancy, with serious implications for maternal and fetal health. The complex interaction between KIR receptors and HLA-C molecules in maternal cells plays a critical role in the condition’s development. Studies have highlighted that variations in KIR genes can influence the risk and progression of PE, with some activating and inhibitory genes appearing to have protective or predisposing effects in specific populations. These findings underscore the importance of further research to fully understand the immunological mechanisms underlying PE and develop targeted diagnostic and therapeutic strategies.

### 8.9. Takayasu’s Arteritis

Takayasu’s arteritis (TA) is a chronic inflammatory vasculitis that affects medium- and large-sized blood vessels, mainly the aorta and its branches. This condition is characterized by inflammation, stenosis, and sometimes occlusion of affected blood vessels, leading to reduced blood flow and potential complications such as aneurysms. The exact cause of TA remains unclear, but it is believed to involve an autoimmune response triggered by genetic predisposition and possibly environmental factors [393].

The study on the association between KIR and HLA genes in TA revealed a significant decrease in the frequency of the KIR2DS4 gene in TA patients compared to healthy controls (Table 5). However, no association was found between KIR and HLA genotypes or interactions between these genes and susceptibility to TA [394]. These results suggest that the KIR2DS4 gene may regulate activation and produce cytotoxic mediators of NK cells in patients with TA.

**Table 5:** KIR genes and other disease.

	Predisposing KIRs	Protective KIRs
Acute ischemic stroke	KIR2DL3 [363] KIR2DL4 [363] KIR2DL5B [363] KIR2DS2 [363] KIR2DS4 [363] KIR3DP1 [363]	/
Birdshot chorioretinopathy	KIR2DS2 [365] KIR2DS3 [365] KIR2DS4 [365]	KIR3DL1 with HLA-Bw4I80 [365] KIR2DL1 with HLA-C2 [365]
Endometriosis	KIR3DS1 [367] KIR2DS2 [369]	KIR2DS5 [368]
Familial Mediterranean Fever	KIR3DP1*003 [371] KIR2DS2 [368]	/
Gaucher Disease	/	KIR2DS2 [180,376] KIR2DL2 with HLA-C1 [180,376]
Paroxysmal nocturnal hemoglobinuria	KIR3DL1 with HLA-Bw4 [379]	/
Polycystic ovary syndrome	KIR3DS1 with HLA-Bw4 [381]	/
Preeclampsia	KIR2DL5 [383] KIR2DL1 [390,391,392] KIR3DL2 [387] KIR2DS1 [388]	KIR2DL1 [387,389] KIR2DS5 [389,390] KIR2DL4 [384,385] KIR2DS2 [390] KIR2DS3 [390]
Takayasu Arteritis	/	KIR2DS4 [394]
Xeroderma pigmentosum	/	/

### 8.10. Xeroderma Pigmentosum

Xeroderma pigmentosum (XP) is a rare genetic disorder characterized by an extreme sensitivity to ultraviolet (UV) radiation, primarily affecting the skin, eyes, and nervous system [395]. Individuals with XP have defects in their DNA repair mechanisms, specifically, in repairing damage caused by UV radiation. As a result, even minimal sun exposure can lead to severe skin burning, early freckling, abnormal skin pigmentation, and a high risk of developing skin cancers at an early age. XP patients may develop eye issues, neurodegeneration, and increased susceptibility to tumors in the CNS. An important aspect of understanding XP’s pathogenesis includes studying HLA. One study only examined the HLA genes (HLA-A and HLA-B) and their association with XP [396] (Table 5). The results showed no significant differences in the frequencies of HLA alleles between the relatives of the patients and a control group. However, there is a higher number of identical HLA alleles among affected siblings, suggesting that some forms of XP may be linked to specific HLA genes. However, the study does not detail which HLA genes are involved. Currently, there are no studies on the association between KIR and XP. Future research is needed to understand how KIR might be involved in its pathogenesis.

## 9. Conclusions

The diversity and complexity of KIRs make them pivotal players in regulating immune responses, with significant implications for health and disease. Their interaction with HLA class I molecules determines the immune activation and inhibition balance, influencing susceptibility to various diseases.

The high degree of variability in KIR genes, combined with their independent inheritance from HLA ligands, contributes to individual differences in immune responses. These genetic variations influence disease susceptibility and progression by altering the inhibitory-to-activating KIR gene ratio or affecting the binding affinity between KIRs and HLA ligands. For instance, genotypes predominately activating KIRs have been linked to increased risk for autoimmune diseases and inflammatory conditions, as they can tip the immune system towards hyperactivation. Conversely, genotypes with higher inhibitory KIRs may promote tolerance but could also impair immune responses against infections and tumors.

However, despite the recognized importance of KIRs, several controversies and knowledge gaps persist in understanding the precise role of KIR polymorphisms in disease susceptibility and progression. Numerous studies have reported associations between specific KIR genotypes and diseases, but results are often inconsistent across different populations and studies. For example, KIR2DS1 has been linked to protection and risk in multiple sclerosis across different cohorts. This could result from ethnic variation in allele frequency and HLA backgrounds, small sample sizes or underpowered studies, and environmental and epigenetic factors not being accounted for.

The balance between activating and inhibitory KIRs is hypothesized to determine disease outcomes, but this binary classification may be oversimplified. Activating KIRs (like KIR2DS1) are generally considered protective in infections but potentially pathogenic in autoimmune conditions. Inhibitory KIRs (like KIR2DL1) are linked to immune tolerance but may impair anti-tumor immunity. This functional dichotomy does not always hold, mainly due to incomplete knowledge of ligand binding for some KIRs and context-dependent receptor signaling influenced by other immune components. Finally, the role of inhibitory vs. activating KIRs in several disease pathogeneses remains speculative, without precise mechanistic data, especially in cancer.

Several gaps in understanding KIR Polymorphisms must be answered, including ligand specificity and functionality, epistasis with HLA and other immune genes, and the impact of copy number variations (CNVs). Indeed, KIR genes show CNVs, which influence receptor expression levels. However, most studies do not differentiate between gene presence/absence and copy number, possibly missing important insights. Finally, environmental triggers, including microbiota composition, modulate NK-cell activity and may interact with the KIR genotype, but these interactions are poorly characterized.

Advances in our understanding of KIR polymorphisms, their interactions with HLA molecules, and their roles in various pathological states hold immense potential for clinical applications.

KIR-based therapies are an exciting frontier in immunomodulation. Several strategies Targeting KIRs may be employed.

Monoclonal antibodies against inhibitory KIRs can block inhibitory signals to enhance NK-cell or CD8+ T-cell cytotoxicity [397]. For example, Lirilumab (IPH2102) blocks *KIR2DL1/2DL2/2DL3* and boosts NK-cell anti-tumor activity. It prevents inhibitory KIRs from binding to HLA-C ligands, allowing NK cells to stay activated [398].

Agonists of inhibitory KIRs promote inhibitory KIR signaling to dampen inflammation in autoimmune diseases. It is based on using agonistic antibodies or ligand mimetics that engage inhibitory KIRs and reduce NK/T-cell activation. In IBD, where overactive NK or CD8+ T cells damage gut epithelium, activating inhibitory KIRs (e.g., *KIR2DL1*, *KIR3DL1*) could restore balance [399].

Blocking activating KIRs prevents excessive activation of NK/T cells driven by activating KIRs (e.g., *KIR2DS1*, *KIR3DS1*). In this context, antibodies or small molecules can be developed to block ligand binding sites of activating KIRs, reducing immune overreaction. They could prevent patients’ excessive epithelial damage and cytokine release, activating KIR-HLA genotypes. Finally, KIRs have been targeted to improve chimeric antigen receptor (CAR) -NK function. CAR-NK cells represent an emerging class of cell-based immunotherapy that offers distinct advantages over CAR-T cells, including a lower risk of graft-versus-host disease (GvHD), cytokine release syndrome (CRS), and neurotoxicity [400,401]. A key aspect of optimizing CAR-NK therapies lies in understanding and manipulating KIRs, which naturally regulate NK-cell function. Inhibitory KIRs interact with HLA class I molecules to prevent NK cells from attacking healthy cells. While critical for self-tolerance, this mechanism can dampen CAR-NK cells’ activity in the tumor microenvironment [400]. Recent studies have shown that modulating inhibitory KIR signaling can enhance CAR-NK efficacy. For example, blocking or genetically editing inhibitory KIRs improves NK-cell persistence and cytotoxicity [400]. Additionally, cytokine stimulation with IL-12, IL-15, and IL-18 has been shown to downregulate inhibitory KIR expression and boost CAR-NK-cell function in vitro and in vivo [402].

There is also growing interest in converting inhibitory signals into activating ones, such as by engineering KIR extracellular domains with activating intracellular motifs (e.g., PD-1/CD28 chimeras). This strategy has been shown to enhance CAR-T efficacy and is potentially translatable to CAR-NK systems [403].

On the other hand, activating KIRs bind to stress ligands or non-classical MHC molecules and promote NK-cell activation. These receptors signal through adaptor proteins like DAP-12, triggering cytotoxic responses [404]. The genetic diversity of KIRs results in varying levels of activating vs. inhibitory receptors among individuals. Notably, the KIR B haplotype, enriched in activating KIRs, has been associated with improved outcomes in hematologic malignancies and is now being considered in donor selection for allogeneic NK-cell therapy (e.g., clinical trial NCT04673617).

Enhancing CAR-NK function using activating KIRs can involve NK-cell selection from KIR B haplotype donors or engineering CAR constructs to incorporate activating KIR domains, such as KIR2DS4 [405]. However, targeting activating KIRs with antibodies or synthetic ligands is complicated by their high sequence similarity to inhibitory KIRs. Novel approaches are under preclinical investigation, including peptide:MHC-based DNA vaccines specific for activating KIRs (e.g., KIR2DS2) [406].

Harnessing the regulatory power of KIRs offers a promising path to improving CAR-NK therapies. Future directions include fine-tuning KIR signaling through gene editing, cytokine priming, or synthetic receptor design and optimizing donor selection based on KIR haplotypes. As the field progresses, integrating KIR biology with CAR engineering could unlock new levels of precision and potency in NK-cell-based immunotherapies.

In conclusion, integrating KIR genotyping into diagnostic and therapeutic strategies could enhance disease management, improve transplant outcomes, and pave the way for novel immunotherapeutic approaches. By leveraging KIRs’ complexity, we can unlock new avenues for understanding and treating diseases.
